# Healthcare provider knowledge, beliefs, and attitudes regarding opioids for chronic non-cancer pain in North America prior to the emergence of COVID-19: A systematic review of qualitative research

**DOI:** 10.1080/24740527.2022.2156331

**Published:** 2023-02-17

**Authors:** Louise V. Bell, Sarah F. Fitzgerald, David Flusk, Patricia A. Poulin, Joshua A. Rash

**Affiliations:** a Department of Psychology, University of New Brunswick, Fredericton, New Brunswick, Canada; b Department of Psychology, Memorial University of Newfoundland, St. John’s, Newfoundland and Labrador, Canada; c Discipline of Anesthesia, Memorial University of Newfoundland, St. John’s, Newfoundland and Labrador, Canada; d Clinical Epidemiology Program, The Ottawa Hospital Research Institute, Ottawa, Ontario, Canada; e Department of Psychology, The Ottawa Hospital, Ottawa, Ontario, Canada; f Department of Anesthesiology and Pain Medicine, Faculty of Medicine, University of Ottawa, Ottawa, Ontario, Canada

**Keywords:** chronic pain management, opioids, opioid prescribing, systematic review, qualitative synthesis

## Abstract

**Background:**

Balance between benefits and harms of using opioids for the management of chronic noncancer pain (CNCP) must be carefully considered on a case-by-case basis. There is no one-size-fits-all approach that can be executed by prescribers and clinicians when considering this therapy.

**Aim:**

The aim of this study was to identify barriers and facilitators for prescribing opioids for CNCP through a systematic review of qualitative literature.

**Methods:**

Six databases were searched from inception to June 2019 for qualitative studies reporting on provider knowledge, attitudes, beliefs, or practices pertaining to prescribing opioids for CNCP in North America. Data were extracted, risk of bias was rated, and confidence in evidence was graded.

**Results:**

Twenty-seven studies reporting data from 599 health care providers were included. Ten themes emerged that influenced clinical decision making when prescribing opioids. Providers were more comfortable to prescribe opioids when (1) patients were actively engaged in pain self-management, (2) clear institutional prescribing policies were present and prescription drug monitoring programs were used, (3) long-standing relationships and strong therapeutic alliance were present, and (4) interprofessional supports were available. Factors that reduced likelihood of prescribing opioids included (1) uncertainty toward subjectivity of pain and efficacy of opioids, (2) concern for the patient (e.g., adverse effects) and community (i.e., diversion), (3) previous negative experiences (e.g., receiving threats), (4) difficulty enacting guidelines, and (5) organizational barriers (e.g., insufficient appointment duration and lengthy documentation).

**Conclusions:**

Understanding barriers and facilitators that influence opioid-prescribing practices offers insight into modifiable targets for interventions that can support providers in delivering care consistent with practice guidelines.

## RÉSUMÉ

**Contexte**: L’équilibre entre les avantages et les inconvénients de l’utilisation d’opioïdes pour la prise en charge de la douleur chronique non cancéreuse (CNCP) doit être soigneusement examiné au cas par cas. Il n’existe pas d’approche uniforme pouvant être adoptée par les prescripteurs et les cliniciens lorsqu’ils envisagent cette thérapie.

**Objectif**: L’objectif de cette étude était de recenser les obstacles et les facilitateurs pour la prescription d’opioïdes pour la douleur chronique non cancéreuse par une revue systématique de la littérature qualitative.

**Méthodes**: Six bases de données ont été consultées pour la période allant de leur création jusqu’en juin 2019 afin d’y repérer les rapports d’études qualitatives sur les connaissances, les attitudes, les croyances ou les pratiques des prestataires en matière de prescription d’opioïdes pour la douleur chronique non cancéreuse en Amérique du Nord. Les données ont été extraites, le risque de biais a été évalué et la confiance envers les données probantes a été notée.

**Résultats**: Vingt-sept études faisant état de données provenant de 599 prestataires de soins de santé ont été incluses. Dix thèmes influençant la prise de décision clinique lors de la prescription d’opioïdes ont émergé. Les prestataires étaient plus à l’aise pour prescrire des opioïdes lorsque (1) les patients étaient activement engagés dans la prise en charge de la douleur, (2) des politiques de prescription institutionnelles claires et des programmes de surveillance des médicaments d’ordonnance étaient en place, (3) des relations de longue date et une alliance thérapeutique forte étaient présentes, et (4) du soutien interprofessionnel était disponible. Les facteurs qui réduisaient la probabilité de la prescription d’opioïdes comprenaient (1) l’incertitude à l’égard de la subjectivité de la douleur et de l’efficacité des opioïdes, (2) une préoccupation pour le patient (p. ex., effets indésirables) et la collectivité (p. ex., détournement), (3) des expériences négatives antérieures (p. ex., recevoir des menaces), (4) des difficultés à adopter des lignes directrices et (5) des obstacles organisationnels (p. ex., durée insuffisante des rendez-vous et longueur de la documentation).

**Conclusions**: La compréhension des obstacles et des facilitateurs qui influencent les pratiques de prescription d’opioïdes permet d’avoir un aperçu des cibles modifiables pour les interventions qui peuvent aider les prestataires à fournir des soins conformes aux directives de pratique.

## Introduction

Chronic noncancer pain (CNCP) is considered one of the most prevalent, debilitating, and complex medical conditions to manage.^1^ Though estimates vary depending on survey methodology, nationally representative data from North America and Europe indicate that 20% to 30% of adults experience CNCP.^2,3^ Urgency of patient needs, aggressive marketing to promote widespread use, demonstrated effectiveness of opioids for acute pain,^4^ and limited access to alternative therapeutic modalities have perpetuated the use of opioids for the treatment of CNCP.^5^ In the United States, between 1997 and 2011, there was a fourfold increase in the sale of prescription opioids,^6^ fivefold increase in drug treatment admissions for prescription opioids (from ~20,000 to ~120,000),^7^ twofold increase in emergency department visits related to pharmaceutical opioids,^8^ and fourfold increase in opioid-related overdose.^9^

Though commonly prescribed, evidence for the benefits of opioids for CNCP is modest (risk difference of achieving the minimally important difference in pain relief versus placebo is 12%),^10^ and potential risks range from nausea and vomiting to addiction, overdose, and death.^5^ Guidelines for prescribing opioids for CNCP are adopting increasingly conservative recommendations in order to balance potential risks against uncertain evidence for benefit. The most recent guidelines published in the United States^11^ and Canada^12^ have been viewed by some as overly conservative and were criticized for being introduced into health care systems that were ill-equipped to provide effective alternatives, such as nonpharmacological interventions for people who live with CNCP.^13–16^ For example, these guidelines recommend against prescribing for people with mental health issues or young people, which could create inequalities in the receipt of quality care among these populations.^15^

Three out of four individuals who use heroin report that their misuse of narcotics began with prescription opioids.^17^ The opioid-related mortality rate in Canada in 2016 was 7.9 per 100,000 population,^18^ with 1 in every 91 deaths being attributed to opioids. In 2019 the rate of opioid-related mortality per 100,000 increased from 7.9 to 11.9. Providers experience an understandable reluctance to prescribe opioids to patients with CNCP given the impact of substance misuse; however, strict opioid prescribing due to concerns over substance misuse can also lead to patient harms (e.g., increased pain, risk of depression, and decreased quality of life).^15^ Patients whose pain is not managed because of underprescribing are also more likely to turn to drug diversion or obtaining illicit opioids in order to relieve pain, placing them at an increased risk of potential overdose.^15^ Health care providers express understandable uncertainty regarding whether, when, and how to prescribe opioids for CNCP given the trade-off between risks and benefits and conflicting evidence on the impact of patients from prescribing or withholding opioids.

It is worth noting that during the first year of the pandemic, there was a 95% increase in apparent opioid toxicity deaths (April 2020–March 2021, 7224 deaths) compared to the year before (April 2019–March 2020, 3711 deaths).^19^ Several factors may have contributed to a worsening of the overdose crisis over the course of the pandemic, including the increasingly toxic drug supply, increased feelings of isolation, stress and anxiety, accessibility of services for people living with pain, delays in medical care for people living with pain, and reduced access to harm services for people who use drugs. Though it is evident that COVID-19 had an impact on opioid-related deaths and a potential impact on prescribing practices, it is important to synthesize evidence pertaining to provider attitudes toward prescribing opioids for CNCP before the pandemic as we transition into the endemic phase of the COVID-19 crisis. Such knowledge can serve as a starting point for understanding the barriers and facilitators of opioid prescribing independent of the significant impact of a global pandemic.

It is important to gain a rich understanding of the barriers and facilitators associated with prescribing opioids for CNCP given that such knowledge is vital for (1) understanding behavior, (2) developing behavioral interventions, and (3) implementing sustainable behavior change in a manner that improves the alignment between evidence and practice. One qualitative synthesis has been published that explored provider experiences of prescribing opioids for CNCP but did not focus on gaining a rich understanding of barriers or facilitators that could be used to develop and enact interventions to modify prescribing practices.^20^ The purpose of this review was to address this gap in the literature by constructing an understanding of barriers and facilitators for prescribing opioids for CNCP through a systematic review of qualitative literature reporting on opioid prescribing for CNCP. Further, it is important to have a synthesis of literature published before the COVID-19 pandemic given that such knowledge is tantamount to understanding changes that may have occurred as a result of the global pandemic.

## Materials and Methods

### Search Strategy

This review is reported in accordance with the Preferred Reporting Items for Systematic Review and Meta-Analysis^21^ and Enhancing Transparency in Reporting of Synthesis of Qualitative Research.^22^ The protocol for this review was preregistered (PROSPERO #CRD42018091640) and published.^23^ Our search strategy was developed by an information specialist in consultation with content experts and subject to peer review by an independent information specialist. A systematic search of CINAHL, Embase, MEDLINE, PsycINFO, and Cochrane CENTRAL was performed from inception to June 3, 2019. We searched the Cochrane Library databases and the Joanna Briggs Institute for relevant systematic reviews and PROSPERO for relevant registered protocols. Manual searches of the reference lists of relevant reviews were performed to identify additional studies.

### Eligibility Criteria

This review considered studies that reported on health care provider attitudes, beliefs, experiences, and perspectives about prescribing opioids to manage CNCP or adhere to recommendations made by clinical practice guidelines. Studies conducted on health care professionals with privilege to prescribe opioids were eligible (e.g., physician, nurse practitioner, physician assistant), including those that reported on medical residents. Studies were excluded if perspectives of the health care professional and patient could not be segregated for synthesis.

Studies that used qualitative data collection and analysis methods and qualitative components of mixed methods studies were eligible, with conference abstracts and review articles excluded. Studies that were conducted in primary or specialist care within North America were eligible, given that opioid-related prescribing practices and nonmedical use and harms are greatest in this area of the world.^24^ Studies were excluded if their primary focus pertained to (1) acute pain, (2) emergent medicine or prescribing within the emergency department, and (3) the management of problematic opioid use.

#### Screening and Selection

Two teams of reviewers independently screened titles and abstracts for eligibility and reviewed full-text publications of all potentially relevant articles using the reference management software Rayyan.^25^ Disagreements on inclusion and exclusion of articles between reviewers were resolved by consensus or arbitration if necessary. Interrater agreement was quantified using Cohen’s kappa and percentage agreement.

### Data Analysis

#### Approach to Evidence Synthesis

A metasynthesis approach was used to systematically identify and synthesize unique qualitative insights from health care professionals. Metasynthesis is a deductive and inductive process with an epistemological basis in objective realism and involves identifying findings, grouping findings into categories, and then grouping categories into synthesized findings.^26^ Following the Thomas and Harden approach to qualitative evidence synthesis,^27^ all text within primary studies was reviewed and coded independently by two reviewers (J.A.R., L.V.B.) to develop descriptive themes.

Meetings were held to review descriptive themes and generate analytical themes until saturation of themes was reached. Analytical themes were coded as barriers or facilitators to prescribing opioids for CNCP according to the theoretical domains framework (TDF).^28^ The TDF distills 33 theories of behavior change into 14 domains that provide a theoretical lens through which to view cognitive, affective, social, and environmental influences on behavior.^28^ Health care provider statements about barriers and facilitators were assigned to a relevant TDF domain, or set of domains, using a process described by Cane et al.^29^ Two reviewers (S.F.F., L.V.B.) independently categorized barriers and facilitators. Discrepancies were resolved through consensus following meetings between team members. Barriers and facilitators of similarity were grouped into themes and agreed upon by consensus of three members (J.A.R., L.V.B., and S.F.F.). The result was a framework of 10 themes and 15 subthemes.

#### Quality Appraisal

Methodological limitations were appraised using the ten-item Critical Appraisal Skills Programme (CASP^30^) tool. The CASP tool is the most commonly used tool in qualitative evidence syntheses in Cochrane and the World Health Organization guideline development processes.^31^ Quality appraisal was performed in duplicate by three reviewers (L.V.B., J.A.R., and S.F.F.) who met and resolved discrepancies through discussion. Study quality was rated as low (met criteria for less than two domains), moderate (met criteria for two domains), and high (met criteria for three or more domains) depending on whether they met four core domains: (1) qualitative methodology was appropriate, (2) recruitment strategy was appropriate for research aims, (3) relationship between researcher and participants was considered, and (4) data analysis was sufficiently rigorous. These four domains were recommended as best capturing the strengths and limitations of qualitative research.^31^

#### Assessing Confidence in Evidence

As recommended by the Cochrane Qualitative and Implementation Methods Group,^31^ confidence in findings was assessed with the Grading of Recommendations, Assessment, Development, and Evaluation-Confidence in the Evidence from Qualitative Reviews (GRADE-CERQual) approach.^32^ Four components were considered: (1) methodological limitations of included studies supporting a review finding, (2) the relevance of the included studies to the review finding, (3) coherence of the review finding, and (4) adequacy of the data contributing to a review result. Confidence ratings started at “high” and were downgraded if concerns were present.

## Results

### Study Identification and Selection

**Figure 1.**
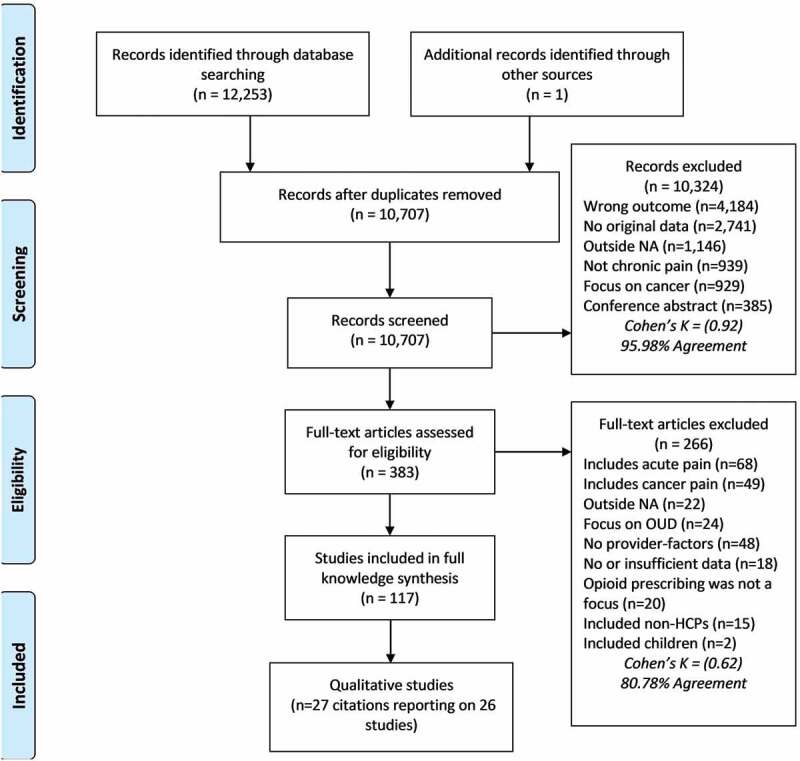
Flow diagram depicting article inclusion and exclusion along with reasons.

We identified 12,253 citations and reviewed 383 full-text articles. Interrater agreement was almost perfect at the title/abstract stage, *k *= 0.92 (95.98%) and moderate at the full-inclusion stage, *k *= 0.62 (80.78%). Twenty-seven qualitative studies^33 − 59^ met inclusion criteria. Two studies reported on the same data,^58,59^ resulting in 26 unique studies. Figure 1 presents a flow diagram depicting inclusions and exclusions of citations reviewed.

Characteristics of included studies are depicted in Table 1. Eligible studies enrolled 599 health care providers (487 primary care providers, 19 specialist physicians, 35 nurse practitioners, 27 pharmacists, 13 physician assistants, 12 medical residents, and 6 reported as “other”).

**Table 1.** Characteristics of included studies.

First author (year)	Provider characteristics	Objective of interview	Type of interview/qualitative analysis	Constructs	Themes relating to opioid prescribing
Barry (2010)	*N* = 23 Internal medicine (*n* = 10), infectious disease (*n* = 4), addiction medicine (*n* = 3), psychiatry (*n* = 2), FM (*n* = 1), did not report (*n* = 3) Incentive: None	Examine attitudes and experiences about treating CNCP	Semistructured/grounded theory	Self-efficacy managing pain with opioids; concerns for society (diversion); short appointment duration; alternative resources; patient–provider relationship	1. Absence of objective or physiological measures of pain impedes pain management (refer to subtheme 1.1) 2. Concern over drug diversion reduces the patient–provider trust (refer to subtheme 2.2) 3. Providers have a difficult time challenging patients in fear of rupturing the patient–provider alliance (refer to theme 4) 4. Limited alternative resources (i.e., specialty pain management clinics) resulted in prescribing opioids to alleviate pain (refer to subtheme 5.1) 5. Providers noted that there was too little time to adequately assess multifactorial nature of pain and complete paperwork (refer to subtheme 5.2) 6. Providers used toxicology screening to support their decision to discontinue opioid prescriptions (refer to theme 9) 7. Providers questioned the motivation for some patients’ requests for opioids for their pain (refer to subtheme 10.1)
Buchman (2016)	*N* = 6 FM (*n* = 1), internal medicine (*n* = 2), psychiatry (*n* = 3) Incentive: None	Examine how adults with CNCP negotiate trust and demonstrate trustworthiness with clinicians	Semistructured/grounded theory	Fear of professional sanction or liability; patient–provider relationship	1. Providers feel that if there is something that objectively describes pain, it changes their approach to their patient (refer to subtheme 1.1) 2. The ultimate fear for providers was for the safety of their patients and society (refer to subtheme 2.1) 3. The potential harm that could occur from the patient diverting opioids was considered distressing (refer to subtheme 2.2) 4. Prior negative experiences with patients bias future interactions (refer to subtheme 3.2) 5. Preexisting positive relationships counteract the antagonistic attitude (refer to subtheme 7.2) 6. Risks associated with prescribing opioids provide a defensible position of distrust and adoption of an “investigator” role that impacts the development of a patient–provider alliance (refer to theme 4 and subtheme 10.1)7. Observing colleagues lose jobs over prescribing results in fear of opioid prescribing and impacts their prescribing behavior (refer to subtheme 10.2)
Calcaterra (2016)	*N* = 25 GP (*n* = 25) Incentive: None	To understand physicians’ attitudes, beliefs, and practices toward opioid prescribing during hospitalization and discharge	Semistructured; open-ended/mixed inductive and deductive approach	Fear for patients; institutional pressure; patient-related salient events; self-efficacy managing pain with opioids	1. Perceived self-efficacy to appropriately prescribe opioids is lower when the objective pain is lacking (refer to subtheme 1.1) 2. Physicians were concerned that by increasing the amount of opioids, they may inadvertently be contributing to opioid dependence or addiction (refer to subtheme 2.1) 3. Physicians expressed concern surrounding opioid diversion to supplement income as some patients had perceived limited resources (refer to subtheme 2.2) 4. Salient negative events (e.g., overdose) bias future prescribing practices for physicians, which results in developing strategies in attempt to prevent such events (refer to subtheme 3.2) 5. Physicians reported little opioid-specific training during residence. As such, opioid prescribing was shaped by their previous experiences (refer to subtheme 5.3) 6. The ability to verify prescription history in PDMPs improves comfort in prescribing opioids (refer to theme 9)
Chang (2017)	*N* = 23 GP (*n* = 18), NP (*n* = 4), PA (*n* = 1) Incentive: $50 gift card	Capture experiences interpreting and implementing guideline recommendations for patients with CNCP and substance use	Semistructured/content analysis method	Alternative resources; patient–provider relationship; patient–provider communication	1. Management plans and UTSs were useful tools to facilitate patient–provider communication about the expectations and risks of opioid therapy (refer to theme 9)
Chang (2016)	*N* = 12 Anesthesia (*n* = 6), GP (*n* = 5), surgeon (*n* = 1) Incentive: None	Assess experiences, perspectives, and attitudes toward the Canadian opioid guideline, elicit barriers and facilitators	Semistructured interview guide with open-ended questions/thematic analysis of verbatim transcripts	Patient–provider communication; education	1. Providers were concerned that it is too easy to turn to opioids to manage CNCP and were concerned about the opioid-related harms for their patients (e.g., dependence; refer to subtheme 2.1) 2. The use of guidelines helps to improve patient–provider communication (refer to subtheme **7**.1) 3. Some providers felt that the guidelines were helpful for decision-making processes, whereas others felt that the volume of information deters updating of prescribing guidelines (refer to theme 11)
Click (2018)	*N* = 32 GP (*n* = 22), osteopath (*n* = 2), NP (*n* = 4), pharmacist (*n* = 2), other (*n* = 2) Incentive: None	Determine what factors lead to prescribing controlled drugs for CNCP through the use of focus groups	Semistructured/iterative process; transcripts combined and compared analysis of transcripts	Institutional pressure; provider-related salient events; patient–provider relationship	1. Providers are reluctant to prescribe opioids due to significant issues of abuse (refer to subtheme 2.1) 2. Providers recall previous negative events and are concerned for their and their staff’s safety when prescribing opioids (refer to subtheme 3.1) 3. Access to alternative therapies is difficult for various reasons (e.g., uninsured patients; refer to subtheme 5.1) 4. Providers report a higher level of comfort prescribing opioids when they have a good and/or long-standing relationship with their patient (refer to subtheme 7.2) 5. Treatment agreements are beneficial for setting boundaries, rules, and standards with patients (refer to theme 9)
Desveaux (2019)	*N* = 22 FM (*n* = 22) Incentive: None	Understand perceived barriers and facilitators to guideline-adherent opioid prescribing	Semistructured/content analysis	Patient–provider relationship; self-efficacy managing pain with opioids; fear for patients; short appointment duration; alternative resources	1. Providers struggled with the objectivity versus subjectivity of pain. Objective results helped make the diagnosis more clear, but subjective results complicated the ability to establish a diagnosis (refer to subtheme 1.1) 2. Providers expressed concern that de-prescribing opioids could lead to an increase in use of stimulants or alcohol (refer to subtheme 2.1) 3. Discussions over opioid prescribing adversely impact the working relationship, subsequently impacting the decision to discuss opioids (refer to theme 4) 4. Lack of accessible nonpharmacological options related to the perceived inability to manage pain (refer to subtheme 5.1) 10. Time constraints in family practice create a barrier for providers to appropriately educate patient about pain and opioids (refer to subtheme 5.2) 5. Having strong communication with the patient and a trusting relationship improves confidence prescribing opioids (refer to subtheme 7.1) 6. Running a practice where patients are well known reduces concerns surrounding prescribing opioids (refer to subtheme 7.2) 7. Providers used tools to help monitor opioid prescribing (i.e., contracts, random urine drug screening; refer to theme 9) 8. Challenging experiences with previous patients led providers to experience fear when patients requested opioids during appointments (refer to subtheme 10.1) 9. Guidelines were helpful summaries of evidence but did not provide tangible tools to assist with prescribing or monitoring practice (refer to theme 11)
Elder (2012)	*N* = 18 GP (*n* = 10), PA (*n* = 8) Incentive: None	Understand differences in pain management among adults with CNCP who are and are not prescribed opioids	Semistructured/transcribed and entered into NVivo 8 qualitative software	Patient–provider relationship; self-efficacy managing pain with opioids	1. Providers find that communication with patients about drug screens/contracts is intimidating (refer to theme 4) 2. Providers noted insufficient time to discuss important patient-related information that arose during conversations between patients and medical assistants (refer to subtheme 5.2) 3. Patients are more forthcoming in conversations over opioids when there is a long-standing relationship (refer to subtheme 7.2) 4. Providers reported benefit from the use of tools to help with drug monitoring (e.g., urine drug screening, using contracts; refer to theme 9) 5. Distrust, skepticism, and suspicion of patient motives increase ambivalence toward prescribing opioids (refer to subtheme 10.1)
Esquibel (2014)	*N* = 16 Resident physician (*n* = 10), attending physician (*n* = 6) Incentive: None	Understand the effects of COT on the doctor–patient relationship	Semistructured/multistep iterative approach; immersion/crystallization process	Fear for patients; patient–provider relationship	1. Providers express difficulty addressing, evaluating, and caring for the patient when the pain is subjective (refer to subtheme 1.1) 2. Providers have concerns over lack of evidence of providing relief and improving function (refer to subtheme 1.2) 3. Providers have concerns over consequences of addiction from prescribing opioids (refer to subtheme 2.1) 4. Providers note that it is easier to prescribe opioids to patients who report using opioids to engage in pain self-management (e.g., exercise; refer to theme 6) 5. Patients who are straightforward and have honest communication facilitated continued opioid prescribing (refer to subtheme 7.1) 6. Providers adopt a defensive role whereby they feel compelled to list concrete reasons for prescribing (e.g., terminal illness, poor prognosis; refer to subtheme 10.1)
Fischer (2017)	*N* = 96 GP (*n* = 96) Incentive: $200	Assess effect of patient requests for specific opioid pain medication on suspected drug seeking and prescribing practices	Semistructured/thematic content analysis; interviews coded quantitatively and analyzed statistically	Fear for patients; patient–provider relationship	1. Knowing the patient well, knowing their history, and believing that the patient’s symptoms were genuine contributed to decision making regarding opioid prescribing (refer to subtheme 7.2)
Fontana (2008)	*N* = 9 NP (*n* = 9) Incentive: None	Examine social and political factors affecting opioid prescribing for CNCP	Semistructured/dialectical analysis	Concerns for society (diversion); institutional pressure; patient-related salient events; education; fear of professional sanction or liability; fear for patients	1. Likelihood of prescribing opioids decreased significantly when objective indicators or pain were not present (refer to subtheme 1.1) 2. Fear of causing addition impacted prescribing practices (refer to subtheme 2.1) 3. Nurses placed restrictions on patients in efforts to control behavior because they felt responsible to help the government prevent illegal abuse and diversion of drugs (refer to subtheme 2.2) 4. Media coverage of overdose and death due to oxycontin reduced willingness to prescribe (refer to subtheme 3.1) 5. Lack of formal education led to prescribing practices being formulated by practices they saw around them (refer to subtheme 5.3) 6. Providers enter relationship with mistrust and suspicion, especially with patients who present with specific drug seeking characteristics (refer to subtheme 10.1) 7. Nurses felt the need to protect themselves from risk to professional licensure (refer to subtheme 10.2) 8. Nurses will refer out if they believe that complying with patient requests will require them to go off protocol (refer to subtheme 10.3)
Harle (2015)	*N* = 15 FM (*n* = 9), internal medicine (*n* = 6) Incentive: None	Understand the decision to prescribe opioids for CNCP in primary care	Semistructured with funneling approach/iterative, open coding analytic approach	Short appointment duration; patient–provider relationship	1. Providers are less willing to prescribe opioids when the subjective pain is out of proportion to the physical examination of pain (refer to subtheme 1.1) 2. Providers struggle with weighing the potential benefits of opioids versus the risk, such as dishonesty, risk of abusing or misusing opioids (refer to subtheme 2.1) 3. Negative past experiences of patients abusing medication lead providers to avoid prescribing altogether (refer to subtheme 3.2) 4. The high-volume environment of usual care did not permit sufficient appointment times to assess and manage pain amidst other assessments (refer to subtheme 5.2) 5. Physicians prioritize improvement in physical function over improvement in pain and are more willing to prescribe opioids when the goal is to improve function (refer to theme 6) 6. Providers base their trustworthiness of a patient off the long-standing relationship, which helps their decision making for prescribing opioids (refer to subtheme 7.2) 7. Providers noted the importance of identifying red flags that might indicate a patient is medication seeking (refer to subtheme 10.1)
Hulen (2018)	*N* = 6 Attending physician (*n* = 3), medical residents (*n* = 2), health psychologist (*n* = 1) Incentive: None	To identify sources of provider stress about prescribing opioids to manage CNCP	Semistructured/exploratory, content-driven approach to applied thematic analysis	Provider-related salient events; alternative resources	1. Providers express difficulty in assessing patients when the pain is subjective (refer to subtheme 1.1) 2. New evidence casting doubt about the effectiveness of opioids and changing guidelines contributed to difficulties surrounding opioid prescribing (refer to subtheme 1.2) 3. Previous salient events contributed to provider reluctance toward opioid prescribing (refer to subtheme 3.1) 4. Providers found conversation related to opioid harms to be fraught with tension, uncertainty, and hopelessness (refer to theme 4) 5. Insufficient appointment duration forced providers to complete risk assessment tools after the appointment, resulting in retrospective bias (refer to subtheme 5.2) 6. Having someone with experience in working with people with CP helped less-experienced providers improve their ability to complete assessments of patient social context (refer to theme 8)
Hurstak (2017)	*N* = 23 GP (*n* = 18), NP (*n* = 4), PA (*n* = 1) Incentive: $50 gift card	Understand perceptions of risk of opioid therapy to improve informed consent process	Semistructured/grounded theory	Fear for patients; fear of professional sanction or liability	1. Providers feared that discontinuing opioids could potentially lead to patients seeking opioids illicitly, potential overdose, misuse, dependence, or diversion. They felt a personal responsibility to prevent this through disciplined prescribing (refer to subtheme 2.1) 2. Providers recognized the harm that can come from diversion (e.g., substance use, violence) and, as such, felt responsible for limiting diversion and diversion-related overdoses (refer to subtheme 2.2) 3. Negative past experiences of patients (e.g., overdose attempts) abusing medication leads providers to avoid prescribing altogether (refer to subtheme 3.2) 4. Providers expressed fear over being responsible for their patient’s death and subsequently losing their license (refer to subtheme 10.2)
Kang (2019)	*N* = 40 Physician (*n* = 15), pharmacist (*n* = 25) Incentive: None	Explore physician and pharmacist perspectives on the opioid crisis and the possibility of physician and community pharmacist collaborations to manage CNCP	Semistructured/applied thematic analysis	Education; alternative resources	1. Providers report the need to have more alternative therapies (refer to subtheme 5.1) 2. Providers felt that they have not been provided sufficient education on managing CNCP in the context of opioid prescribing (refer to subtheme 5.3) 3. Providers relied on PDMP, urine screens, and patient history to mitigate abuse and diversion (refer to theme 9)
Kennedy (2017)	*N* = 40 GP (*n* = 34), NP (*n* = 3), other (*n* = 3) Incentive: Meals	Assess experiences of opioid tapering with patients on long-term opioid therapy	Semistructured in-person focus groups/mixed inductive–deductive method	Fear for patients; goal setting; short appointment durations; alternative resources; patient–provider relationship; patient–provider communication; education	1. Providers struggle to prescribe opioids safely while keeping patients satisfied with their pain care (particularly at higher doses; refer to subtheme 2.1) 2. Providers find conversations about opioid tapering emotionally exhausting, challenging, and draining and will often avoid or postpone having these conversations (refer to theme 4) 3. Providers noted there was not enough time during a clinic visit to discuss tapering and additionally not enough time and resources to support opioid tapering (refer to subtheme 5.2) 4. Providers sought their own training related to managing complex chronic pain, because it was not taught to them during school or residency (refer to subtheme 5.3) 5. Providers reported benefit in discussing unpleasant, common side effects (e.g., low testosterone, erectile dysfunction, memory loss) to help engage patients in opioid tapering (refer to subtheme 7.1) 6. Providers are concerned that patients may not entirely share pain-related symptoms or opioid-related adverse effects for fear that the provider will taper their opioids (refer to subtheme 10.1)
Knight (2017)	*N* = 23 GP (*n* = 18), NP (*n* = 4), PA (*n* = 1) Incentive: None	Explore the educational, clinical, and social factors that contribute to opioid prescribing	Semistructured/iterative process; inductive and deductive coding	Concerns for society (diversion); patient–provider relationship; education; self-efficacy managing pain with opioids	1. Providers expressed reluctancy toward using opioids because they feel there is not enough evidence demonstrating efficacy (refer to subtheme 1.2) 2. Providers were encouraged to evaluate evidence for effectiveness more thoroughly and weigh this against potential dangers (patient safety became a more prominent concern; refer to subtheme 2.1) 3. Concern over drug diversion was a prominent decisional factor and decreased providers’ willingness to prescribe opioids (refer to subtheme 2.2) 4. Providers were concerned that having patients sign PDMP contracts could create a climate of mistrust that might threaten the therapeutic alliance (refer to theme 4) 5. Given that few alternative options were present, providers felt that treating people’s pain with opioids was the right thing to do (refer to subtheme 5.1) 6. Providers felt relief from the introduction of pharmacovigilance measures (UTS, PDMPs, pain agreements) to help with diversion and misuse of prescribed opioids (refer to theme 9) 7. Clinicians describe their education as their responsibility to be attentive, to be nonjudgmental regarding pain, and to believe their patients regardless of the amount of morphine they need instead of adopting a defensive stance (refer to subtheme 10.1) 8. Providers feel supported by clinic policies because they help to depersonalize and legitimize clinicians’ decision-making practices regarding opioids (refer to subtheme 10.3)
Liddy (2017)	*N* = 26 FM (*n* = 25), other (*n* = 1) Incentive: None	Identify themes emerging from exchanges between PCPs and specialists regarding patients with chronic pain	eConsult service between PCP and specialist/thematic analysis of cases using a constant comparison approach	Self-efficacy; alternative resources	1. PCPs sought advice from pain specialists about treatment strategies (refer to theme 8)
Militello (2018)	*N* = 10 PCP (*n* = 10) Incentive: None	Identify patient, social, and provider factors that influence how providers assess and manage CNCP	Critical decision method interviews/thematic analysis using framing and anchors	Education; goal setting	1. Providers expressed uncertainty regarding the efficacy of opioids for people with CNCP (refer to subtheme 1.2) 2. Lack of available alternative nonopioid therapies, housing insecurity, transportation, and social support systems contribute to provider decision making for treatments for patients (refer to subtheme 5.1) 3. Some providers note that urine drug tests do not detect all relevant substances and instead think that a blood test may better meet the spirit of the guidelines (refer to theme 9) 4. Providers feel that guidelines are diffuse and require interpretation and that they may not be able to meet recommended guidelines in their clinic (refer to theme 11)
Penney (2017)	*N* = 25 GP (*n* = 25) Incentive: None	Identify practical issues patients and providers face when assessing alternatives to opioids	Structured; focus group/iterative process using inductive and deductive coding	Short appointment duration; alternative resources; self-efficacy; managing pain with opioids	1. Almost all providers framed opioids as problematic because of the risk of dependence and believed that they would not be able to get patients off of opioids (refer to subtheme 2.1) 2. Providers report difficulty treating patients with narcotic drugs because of the challenges that come along (i.e., diversion; refer to subtheme 2.2) 3. Providers feared that suggesting changing prescriptions would harm the rapport with their patients when they were resistant to trying anything other than opioids (refer to theme 4) 4. Despite efforts to discourage the use of opioids, providers felt that this was often the only option available (refer to subtheme 5.1) 5. Providers report that having 10 min per patient is an inadequate amount of time to explore things other than drugs for people with chronic pain (refer to subtheme 5.2) 6. Providers liked using the health plan that was introduced because it includes urine drug screening and implementation and signing of written opioid therapy plans that helped to reduce and better manage the use of opioid medications (refer to theme 9)
Robertson (2014)	*N* = 6 FM (*n* = 5), other (*n* = 1) Incentive: None	To understand the impact of a point-of-care opioid tool called Opioid Manager on clinical practice	Semistructured/thematic analysis; content analysis; code–recode technique used to verify content validity	Self-efficacy managing pain with opioids; patient–provider communication; education; education on tools	1. Involving the patient in conversations surrounding opioid prescribing facilitated willingness to prescribe (refer to subtheme 7.1) 2. Keeping things open with their patients facilitates a strong therapeutic alliance (refer to subtheme 7.2) 3. Some providers will use opioid management tools on all of their patients to allow the same amount of scrutiny for everyone, whereas others will use it with some patients but not others (depending on the level of risk of the patient; refer to theme 9) 4. Providers likened regulatory scrutiny to tax audits, saying it may be a growth experience, but it is not fun (refer to subtheme 10.2) 5. Providers felt like they were flying by the seat of their pants when prescribing and did not feel like they had any guidelines to go by (refer to theme 11)
Satterwhite (2019)	*N* = 23 Physician, NP, physician assistant Incentive: None	Identify contextual factors that contribute to time scarcity and its effects on quality of care when prescribing opioids in primary care	Semistructured and open-ended/grounded theory	Short appointment durations; alternative resources	1. Providers feel that dealing with pain management is time draining, takes up a significant amount of their schedule, takes time away from other patients, and may take time away from the specific patient to address any other health concerns (e.g., mental health; refer to subtheme 5.2) 2. Establishing functional goals with new or inherited patients being prescribed opioids was viewed as an important method for facilitating a long-term strong working alliance (refer to theme 6)
Spitz (2011)	*N* = 26 Physician (*n* = 23), NP (*n* = 3) Incentive: None	Identify attitudes and perceived barriers and facilitators to prescribing opioids among older adults	Focus group; semistructured/content analysis	Patient-related salient events; patient–provider relationship; fear for patients	1. Providers were skeptical about patients and were reluctant to prescribe opioids when patients’ pain scores did not correspond with their behaviors during the visit (refer to subtheme 1.1) 2. A minority of providers mentioned that opioids are effective when used in the “right” older patient (e.g., one who can understand the regimen and can anticipate the side effects and were not considered front-line treatment; refer to subtheme 1.2) 3. Providers reported the fear of causing harm to older patients (refer to subtheme 2.1) 4. Fear of causing harm to patients was related to previously experienced events (refer to subtheme 3.2) 5. Few physicians were concerned about the possible legal or regulatory sanctions of opioid prescribing. Nurse practitioners reported concern over regulatory scrutiny for writing high-volume scripts for patients when their physician was away from the practice (refer to subtheme 10.2)
Tong (2019)	*N* = 16 PCPs (*n* = 16) Incentive: None	Understand provider factors that inform the use of opioids for CNCP in primary care	Semistructured/template and emergent coding processes	Short appointment duration; alternative resources; institutional pressure	1. Providers note that there are limited alternative therapies for pain management (refer to subtheme 5.1) 2. Providers lack time to appropriately manage chronic opioids (refer to subtheme 5.2) 3. Providers have greater willingness to prescribe when risk of abuse is low and opioids help improve function (e.g., ability to work; refer to theme 6)
Wyse (2018)	*N* = 24 Physicians (*N* = 18), NP (*N* = 4), physician assistant (*N* = 2) Incentive: None	To learn about the methods PCPs used to address prescription opioid misuse and aberrant opioid-related behaviors	Semistructured/content analysis	Alternative resources	1. The mismatch between the needs of patients on long-term opioid therapy evidencing aberrant behaviors or symptoms of opioid use disorder and the substance use disorder treatment offerings that were available was noted by providers (refer to subtheme 5.1) 2. Time constraints were faced because the consent procedure can be lengthy and the consent was located in the electronic medical record, making it difficult to access (refer to subtheme 5.2) 3. Providers would sometimes turn to pharmacists to help design opioid tapers. They also appreciated knowing that they can rely on external resources to share responsibility about opioid tapering (refer to theme and subtheme 10.3)
Wyse (2019)	*N* = 24 Physicians (*N* = 20), NP (*N* = 4) Incentive: None	Identify provider strategies for managing aberrant medication behaviors among patients prescribed chronic opioid therapy	Semistructured/content analysis	Goal setting; short appointment duration; provider-related salient events; patient–provider relationship	1. Providers feel that conversations with patients were unpleasant, with one provider recalling violent reactions from patients when their opioid prescriptions were changed (refer to subtheme 2.1) 2. Clinicians found it difficult to be on the receiving end of complaints regarding their perceived lack of concern for patients’ pain, when they believed that their actions were ultimately in the patients’ best interest (refer to theme 4) 3. Providers report that conversations about opioids were time consuming and appreciated that nurses provided group education visits so they do not have to do this individually with each patient (refer to subtheme 5.2) 4. Providers reported benefit from the use of a treatment agreement when making decisions about restricting opioids following aberrant behaviors (refer to theme 11)

COT = chronic opioid therapy; CPG = clinical practice guideline; ED = emergency department; EMR = emergency medical record; FM = family medicine; GP = general practitioner; LPN = licensed practical nurse; NP = nurse practitioner; PCP = primary care physician; RN = registered nurse; TA = treatment agreement; UDT = urine drug test.

### Methodological Quality Assessment

Supplemental Table 1 provides a summary of the critical appraisal of the quality of included studies. Overall, 6 studies were rated globally as having high quality, 10 of moderate quality, and 9 of low quality. Of note, the researchers’ reflexivity (i.e., critical self-evaluation of the researcher’s position and explicit recognition and acknowledgment of how this position may affect the research process and outcomes^60^) was given insufficient documentation or consideration in a majority of studies (21 of 27). Also of note, almost half of included studies paid insufficient attention to the method, rationale, and discussion of the recruitment process (e.g., who elected not to participate and why) to contextualize the potential for selection bias (12 of 27).

### Synthesis of Evidence

Table 2 depicts a summary of findings and Supplemental Table 2 depicts a summary of qualitative findings and confidence assessments using the CERQual approach. Themes were categorized based on their impact on prescribing (i.e., barriers, facilitators, and mixed). This categorization was done at the level of theme. Results are presented in the following with representative quotes. A list of quotations supporting each theme is provided in Supplemental Table 3.

**Table 2.** Summary of findings.

Theme	Contributing studies	Description of review finding	CERQual assessment	Explanation of CERQual assessment
**Barriers**				
**Theme 1: Uncertainty toward subjectivity of pain and efficacy of opioids led to provider reluctance toward prescribing opioids**				
1.1 Inability to perform an unbiased evaluation of pain and identify objective explanation decreased providers’ willingness to prescribe	^33–35,41,43,44,51–53^	Providers questioned the veracity of self-reported pain in the absence of objective markers, which resulted in discomfort with prescribing opioids	High confidence	Moderate concerns about methodological limitations
1.2 Providers who questioned the perceived efficacy of opioids were reluctant to prescribe	^41,47,51,53,55^	Uncertainty about the effectiveness of providing relief and improved function from opioids for CNCP contributed to difficulties prescribing opioids	Moderate confidence	Moderate concerns about methodological limitations and adequacy
**Theme 2: Concern for patient and community safety resulted in reluctance to prescribe opioids for CNCP**				
2.1 Concern over patient safety resulted in caution when prescribing opioids	^34,35,37,38,39,41,43–47,49,51,52,58^	Providers wanted to do the right thing by helping patients manage pain but were concerned for patient safety (e.g., addiction, overdose), which resulted in caution when prescribing opioids for CNCP	High confidence	Moderate concerns about methodological limitations
2.2 Concern over drug diversion resulted in caution when prescribing opioids	^33–35,43,45,47,49^	Concern over potential adverse effects of drug diversion within the community results in caution when prescribing opioids for CNCP. Examples of potential adverse effects of drug diversion included violence, poor pain management, overdose, and damaging provider–patient trust	Moderate confidence	Serious concerns about methodological limitations. Moderate concerns about adequacy
**Theme 3: Past negative experiences resulted in reluctance to engage in conversations surrounding opioid prescribing**				
3.1 Negative personal-related events decreased willingness to prescribe opioids	^39,43,53,58^	Providers reported a reduced willingness to prescribe opioid analgesics to manage CNCP due to the experience of negative salient personal events, including perceived blame for opioid-related patient overdose deaths and angry or aggressive interactions with patients	Moderate confidence	Serious concerns about methodological limitations. Moderate concerns about adequacy
3.2 Negative patient-related events decreased providers’ willingness to prescribe opioids	^34,35,44,45,51^	Provider’s previous negative patient-related events (e.g., patients misusing opioids or patient death) led to provider biases against prescribing opioids in the future	High confidence	Moderate concerns about methodological limitations
**Theme 4: Providers expressed difficulty having conversations surrounding opioid prescribing and feared these conversations would harm the therapeutic alliance**				
	^33,34,36,40,46,47,49,52,53,58^	Most providers found conversations related to opioid use, harms (e.g., side effects, misuse, hyperalgesia), tapering, and drug screening/contracts to be fraught with tension, uncertainty, and fear of rupturing the patient–provider alliance	Moderate confidence	Serious concerns about methodological limitations
**Theme 5: Organizational barriers such as a lack of opioid alternatives, appointment time constraints, and inadequate medical education limited CNCP management strategies**				
5.1 Unavailability of opioid alternatives facilitated opioid prescribing	^33,39,47,49,52,54,55,57,59^	Most providers reported that insufficient availability of opioid-alternative pain management resources for patients (e.g., specialty pain management services or services focusing on co-occurring addiction and pain management) was positively associated with opioid prescribing	Moderate confidence	Moderate concerns about methodological limitations
5.2 Appointment time constraints limited thoughtful prescribing practices and patient education surrounding opioid treatment	^33,40,44,46,49,52,53,56–59^	Providers reported that brief appointment durations did not provide sufficient time to prescribe opioids, including assessment, education, enacting a treatment agreement, and documentation	Moderate confidence	Serious concerns about methodological limitations
5.3 Inadequate formal education during medical school or residency resulted in unstandardized prescribing practices	^35,43,46,54^	Physicians perceived a lack of formal education on prescribing opioids for the management of CNCP during medical school or residency. Some providers allowed personal experiences and interactions with clients to shape their prescribing habits, whereas others took the initiative to seek continuing medical training	Moderate confidence	Moderate concerns about methodological limitations
**Facilitators**				
**Theme 6: Prioritizing improvement in function increased providers’ willingness to discuss opioid prescribing**				
	^41,44,56,57^	Providers prioritize improvement in function over remittance of pain and reported greater willingness to discuss opioid prescribing and discontinuation when the goal was to improve patient function	Moderate confidence	Moderate concerns about methodological limitations, adequacy, and relevance
**Theme 7: A strong therapeutic alliance increased discussions surrounding and willingness to prescribe opioids**				
7.1 Strong patient–provider communication facilitated open and willful conversations surrounding opioid prescribing	^37,41,46,50,52^	A strong working alliance, candid communication, and shared decision making improved provider willingness and confidence to prescribe and taper opioids for CNCP	Moderate confidence	Moderate concerns about methodological limitations
7.2 Long-standing patient–provider relationship and trust increased willingness to engage in opioid discussions	^34,39,40,42,44,50,52^	Having a long-standing relationship with a patient was viewed as improving trust, communication, working alliance, and subsequent willingness to engage in discussions around prescribing opioids for CNCP	High confidence	Moderate concerns about methodological limitations
**Theme 8: Interprofessional support or mentorship improved provider willingness and confidence to engage in patient discussions surrounding opioid prescribing**				
	^48,53,59^	Mentorship and support from specialists, experienced colleagues, or allied health professionals improved PCP willingness and confidence to engage patients in discussions around prescribing opioids for CNCP.	Low confidence	Serious concerns about methodological limitations, adequacy, and relevance. Moderate concerns about coherence
**Theme 9: Use of PDMPs for risk management contributed to providers’ confidence in opioid prescribing and discontinuation**				
	^33,35,36,39,40,47,49,50,52,54,55,58^	The use of PDMP, UDTs, and patient history were perceived as beneficial to decision making for opioid prescribing, better management of medications, and the prevention of opioid abuse and diversion	Moderate confidence	Moderate concerns about methodological limitations and coherence
**Mixed**				
**Theme 10: External policies and tools increased providers’ confidence in decisions to prescribe or discontinue opioids. Meanwhile, external pressures decreased willingness to prescribe**				
10.1 Providers adopted a defensive stance when prescribing opioids	^33,34,40,41,43,44,46,47,52^	Most providers adopt a position of distrust, or a “defensive stance,” when entering a patient–provider relationship and prescribing opioids. Inconsistencies or red flags (e.g., inconsistency of objective vs. subjective findings) may result in avoiding prescribed opioids altogether	Moderate confidence	No/minor concerns about coherence, adequacy, and relevance. Serious concerns about methodological limitations
10.2 Concern over regulatory scrutiny decreased willingness to prescribe opioids	^34,43,45,50,51^	Concern over regulatory scrutiny or being held liable for a patient overdose or death results in fear and reduces willingness to prescribe opioids for CNCP	Moderate confidence	Moderate concerns about adequacy. Serious concerns about methodological limitations
10.3 Institutional policies reduced subjectivity of prescribing opioids and were perceived as beneficial	^43,47,58,59^	Providers reported that institutional policies around opioid prescribing and tapering set by an organizational opioid safety committee or ethics board reduced subjectivity and increased confidence in decision making	Moderate confidence	Moderate concerns about adequacy. Serious concerns about methodological limitations
**Theme 11: Some providers struggled to enact guidelines, especially among complex patients. Contrarily, some providers felt the guidelines were helpful for making decisions and setting boundaries**				
	^37,50,52,55,58^	Most providers felt that guidelines were difficult to enact and felt like they were “flying by the seat of their pants.” For example, some felt like these guidelines were not straightforward or did not pertain to complex patients. Some providers expressed gratitude for prescribing guidelines, indicating that they enabled helpful decision-making processes and aided in setting effective boundaries	Moderate confidence	Moderate concerns about methodological limitations, coherence, and adequacy

See Table 1 for abbreviations.

## Barriers to Prescribing Opioids

### Theme 1: Uncertainty Toward Subjectivity of Pain and Efficacy of Prescribing Opioids Led to Providers’ Reluctance Toward Opioid Prescribing

#### Subtheme 1.1: Inability to Perform an Unbiased Evaluation of Pain and Identify an Objective Explanation Decreased Providers’ Willingness to Prescribe (GRADE-CERQual Confidence Level: High)

Nine articles^33–35,41,43,44,51–53^ reporting on 148 providers (90 primary care, 12 nurse practitioners, 12 residents, 18 internal medicine, 12 specialists, 1 health psychologist, 3 other) identified difficulty assessing pain in the absence of objective indicators, such as tissue damage.^24–26,31,33,34,41–43^ Providers noted that the subjectivity of pain complicates the ability to establish a diagnosis, leaving discomfort in managing pain and caring for the patient. In the words of one provider:

“Somebody will say, ‘Oh, I have ten out of [ten] pain’, but they’re sitting there, comfortably. They’re walking around, totally fine, and you’re like, ‘This is not what I would consider ten out of ten pain.’”^44^

Willingness to prescribe opioids also decreased when objective indicators of pain were not present:

“If I can’t find the cause of the pain, that is always an interesting problem. The reality is that I would never give somebody narcotics in that case.”^43^

#### Subtheme 1.2: Providers Who Questioned the Perceived Efficacy of Opioids Were Reluctant to Prescribe (GRADE-CERQual Confidence Level: Moderate)

Most providers working within primary care reported uncertainty about the efficacy of opioids for relief of pain and improvement of function. This theme was supported by five articles that reported on 81 providers (60 primary care, 7 nurse practitioners, 1 physician assistant, 12 residents, 1 health psychologist).^41,47,51,53,55^ Some providers indicated that doubts over the efficacy of opioids arose from recent empirical evidence. Uncertainty about the efficacy of opioids was seen as contributing to a reluctance toward prescribing opioids to manage CNCP given the potential for adverse effects:

Do we have evidence to show that these opiates are going to work in chronic nonmalignant pain? You know, no I don’t think so. … The pendulum has kind of moved the other way. We’re trying to be more thoughtful in prescribing. So I think that’ll mean less prescriptions, stopping meds that are not effective or dangerous. Safety has become kind of an issue, a bigger issue as well.^47^

### Theme 2: Concern for Patient and Community Safety Resulted in Reluctance to Prescribe Opioids for CNCP

#### Subtheme 2.1: Concern over Patient Safety Resulted in Caution When Prescribing Opioids (GRADE-CERQual Confidence Level: High)

Many providers wanted to “do the right thing” by helping their patients manage pain by prescribing opioids. However, patient safety was viewed as one of the most important factors when prescribing opioids, and the thought of potentially harming their patients (e.g., fear of causing addiction or overdose) was one of the most salient concerns that resulted in caution with regard to prescribing opioids for CNCP. This theme arose across 14 studies^34,35,37,39,41,43–47,49,51,52,58^ that reported on 252 providers (189 primary care, 27 nurse practitioners, 2 physician assistants, 10 residents, 8 internal medicine, 10 specialists, 2 osteopaths, 2 pharmacists, and 2 others). Several studies within this theme discussed concerns over patient safety when tapering or discontinuing opioids, leading to the decision to continue prescribing while fully aware of and concerned about risks:

“In others, stimulant use and alcohol use goes way up when I titrate down their opioids. So, prescribing opioids in a controlled fashion for their pain, despite their pain risk, seems to be less risky.”^52^

Some studies within this theme indicated that concern over patient safety was partially dependent on patient characteristics, such as age:

I just have a hard time prescribing opioids in my older patients. I get frightened with 80+ year olds; how are they going to respond? Am I going to absolutely drop them to the floor even with a small dose?^51^

I would be very hesitant to start chronic [opiates] in a young person regardless of gender.

It may be that [it is] age more than actual pregnancy status that we’re concerned about.^39^

#### Subtheme 2.2: Concern over Drug Diversion Resulted in Caution When Prescribing Opioids (GRADE-CERQual Confidence Level: Moderate)

Seven articles including 134 providers (88 primary care, 17 nurse practitioners, 2 physician assistants, 12 internal medicine, 12 specialists) discussed concerns over drug diversion when prescribing opioids.^33–35,43,45,47,49^ General practitioners felt that they were contributing to potential drug diversion within their community by prescribing opioids to their patients and reported reluctance to prescribe opioids as a result.

In my first year here, I was very kindly giving benzodiazopenes to a woman for neck spasms and Percocet for her neck pain based on her records and what she told me. And when I found out I was also prescribing for her sister and her mother I realized that single-handedly I was probably prescribing for all of New Haven and immediately got them off.^33^

Concerns over drug diversion were complex and were influenced by the population that frequented the clinics where providers worked. As noted by one provider:

“I think our population can divert quite a few meds. I think their financial situations can be really tenuous. Sometimes they sell pills to survive.”^35^

One provider indicated that the clinical decision-making algorithm favors harms to society under such circumstances:

“The harm to society overrides the patient.”^43^

### Theme 3: Past Negative Experiences Resulted in Reluctance to Engage in Conversations Surrounding Opioid Prescribing

#### Subtheme 3.1: Negative Personal-Related Events Decreased Providers Willingness to Prescribe Opioids (GRADE-CERQual Confidence Level: Moderate)

Four articles elucidated the impact of negative salient personal events on opioid prescribing among a sample of 71 providers (45 primary care, 17 nurse practitioners, 2 residents, 2 osteopaths, 2 pharmacists, 1 health psychologist, 2 other).^39,43,53,58^ Providers reported a reduced willingness to prescribe opioids for CNCP due to the experience of negative salient personal events. Some providers noted that the perceived blame for opioid-related patient overdose or death impacted their future prescribing practices. Others noted that they were not willing to prescribe opioids to their patients who were confrontational, threatening, or violent due to concerns over personal safety:

“I’ve had someone hit me with their cane. I’ve had my car keyed. … I already had someone that wanted to kill me several years ago about this. …”^58^

#### Subtheme 3.2: Negative Patient-Related Events Decreased Providers’ Willingness to Prescribe Opioids (GRADE-CERQual Confidence Level: High)

Five articles discussed the impact of negative patient-related events on prescribing opioids among a sample of 95 providers (78 primary health care, 7 nurse practitioners, 1 physician assistant, 8 internal medicine, 3 specialists, 2 other).^34,35,44,45,51^ Negative patient-related events, such as patients misusing opioids or opioid-related patient deaths, were associated with hesitancy when prescribing opioids. After recalling negative events, providers were more guarded about their prescribing behaviors in order to avoid future patient-related overdose, misuse, or death.

It is both your cumulative experience and, sometimes, when you’ve had a negative experience, it really biases how you think. I’ve had an experience where my patient actually overdosed. She crushed up the oxycodone we were giving her in the hospital and shot it up through her central line and died. We’ve all had experiences with opioids being abused. This just happened to be a very dramatic thing that happened right under my nose. It just makes me more guarded, in terms of my practice and the lengths people will go through to do harm to themselves with opioids.^35^

### Theme 4: Providers Expressed Difficulty Having Conversations Surrounding Opioid Prescribing and Feared These Conversations Would Harm the Therapeutic Alliance (GRADE-CERQual Confidence Level: Moderate)

Ten articles^33,34,36,40,46,47,49,52,53,58^ highlighted the difficult nature of conversations surrounding opioids among a sample of 210 providers (152 primary care, 15 nurse practitioners, 10 physician assistants, 2 residents, 12 internal medicine, 12 specialists, 1 health psychologists, 6 other). Most general practitioners indicated that conversations surrounding opioid-related harms (e.g., side effects, misuse), tapering, and treatment agreements were difficult to broach with clients, fraught with tension, and avoided when possible. Some providers also found that these conversations ruptured the patient–provider relationship.

Those conversations around “I don’t think prescribing this is appropriate” … physicians tend to shy away, because I think they expect them to be confrontational. … You want your patients to trust you, to believe you, you want the clinical encounter to be relatively conflict free. I mean, I think that’s the biggest struggle, and I think some doctors, as a result, don’t prescribe opioids. Which is wrong, because they’re a legitimate clinical and pharmacological resource, but there’s that fear.^52^

### Theme 5: Organizational Barriers Such as a Lack of Opioid Alternatives, Appointment Time Constraints, and Inadequate Medical Education Limited CNCP Management Strategies

#### Subtheme 5.1: Unavailability of Opioid Alternatives Facilitated Opioid Prescribing (GRADE-CERQual Confidence Level: Moderate)

Nine studies described the impact insufficient availability of nonopioid treatments for pain management has on prescribing opioids for CNCP among a sample of 215 providers (147 primary care, 12 nurse practitioners, 3 physician assistants, 10 internal medicine, 9 specialists, 2 osteopath, 27 pharmacists, 5 other).^33,39,47,49,52,54,55,57,59^ Lack of available nonopioid treatments (e.g., specialty pain management services, community resources) was viewed as facilitating the prescription of opioids for CNCP. As noted by one provider:

It takes some time to find a resource, for physio or for massage, all those other things that could help manage pain. And it takes some time to get those in place. There’s not another great alternative pain management mechanism out there, both pharmacological and even, again, nonpharmacological. Often [patients don’t qualify], and they can’t get access to a lot of the other supports.^52^

A perceived lack of alternative approaches to pain management was viewed as interacting with social factors, such as housing insecurity, lack of financial means or insurance, and poor access to transportation or social support, that further contributed to limited accessibility of care. Similarly, patient complexity (e.g., co-occurring substance misuse) and lack of funding to promote nonopioid therapies were viewed as further contributing to the problem.

#### Subtheme 5.2: Appointment Time Constraints Limited Thoughtful Prescribing Practices and Patient Education Surrounding Opioid Treatment (GRADE-CERQual Confidence Level: Moderate)

Information from 11 articles that included 212 providers (138 primary care; 7 nurse practitioners, 10 physician assistants, 2 residents, 16 internal medicine, 9 specialists, 1 health psychologist, 3 other) indicated that time constraints during appointments were a barrier for providers to adequately assess and manage pain.^33,40,44,46,49,52,53,56–59^ Providers noted that brief appointment times did not allow for meaningful conversations surrounding opioid prescribing, which led to inadequate patient education about pain and opioids.

And I think that is tough in our busy practices, to actually take time to really educate people about pain, and that’s why we offer this chronic pain program through our family health team. … But you can imagine a physician that doesn’t have access to that. You don’t generally have the time to spend in sessions talking about how to manage pain.^52^

We get our little ten-minute per patient, which is so grossly, woefully inadequate amount of time to see a patient. Ten minutes, right, for all your problems. And so nobody wants to take the time to explore things other than drugs for people with chronic pain.^49^

The impact of short appointment durations on opioid prescribing was viewed as compounded by the volume of paperwork required when providers prescribe opioids. This led some providers to reduce the number of appointments accepted or limit the number of patients prescribed opioids on their caseload:

That is why you have to limit the number [of patients prescribed opioids], you can’t carry a lot of them because there’s so much paperwork.^33^

So, I heard all this stuff that I’m supposed to be doing, taking a complete history, complete addiction history. … But I don’t have time to do what I am supposed to do in terms of proper treatment, opioid treatment, so I cut corners a bit.^56^

#### Subtheme 5.3: Inadequate Formal Education during Medical School or Residency Resulted in Unstandardized Prescribing Practices (GRADE-CERQual Confidence Level: Moderate)

Four studies reported on the perceived adequacy of formal education surrounding the prescription of opioid analgesics for the management of CNCP among 114 health care providers (74 primary care, 12 nurse practitioners, 25 pharmacists, 3 other).^35,43,46,54^ General practitioners indicated that perceived inadequate education on the prescription of opioids for pain management received during medical school or residency was a barrier to prescribing. As summarized by Kang et al.^54^:

“Many participants felt that there was a lack of provider education on this topic.”

Some providers allowed personal experiences and interactions with clients to shape their prescribing habits, and others took the initiative to seek continuing medical training.

I have had a kidney stone, and if a person has a kidney stone, they get pain medication.^43^

I’ve basically sought out my own training because it was not something that was taught to me in medical school or residency.^46^

## Facilitators of Prescribing Opioids

### Theme 6: Prioritizing Improvement in Function Increased Providers’ Willingness to Discuss Opioid Prescribing (GRADE-CERQual Confidence Level: Moderate)

Primary care providers prioritize improvement in function over reductions in self-reported pain and reported greater willingness to discuss opioid prescribing and discontinuation when the goal was to improve patient function. This theme was supported by four articles^41,44,56,57^ reporting on 70 providers (54 primary care, 6 internal medicine, 23 other.^34,46,47^ For example, one provider indicated the importance of setting functional goals despite the additional time that it takes to do so because this was viewed as a way to maintain a strong long-term working alliance:

In terms of trajectory with patients … either if I’m inheriting them or if I’m starting over with them, or even starting with these new patients, [I try] to establish some functional goals. … I totally do not have time to do any of [it] but it’s not an option [not to]. If you don’t do it the whole relationship ends up being a disaster … because every time you see them you’re just arguing about whether or not it’s [the pain’s] better, whether [opioid medication] makes them better or worse. …^56^

### Theme 7: A Strong Therapeutic Alliance Increased Discussions Surrounding and Willingness to Prescribe Opioids

#### Subtheme 7.1.: Strong Patient–Provider Communication Facilitated Open and Willful Conversations Surrounding Opioid Prescribing (GRADE-CERQual Confidence Level: Moderate)

Five articles highlighted the influence of patient-provider communication on prescribing opioids among a sample of 96 providers (66 primary care, 3 nurse practitioners, 10 residents, 7 specialists, 10 other).^37,41,46,50,52^ Providers felt more confident prescribing opioids for CNCP when they perceived interactions with patients to be straightforward and honest with good two-way communication, highlighting the importance of cultivating a strong working alliance and a shared decision-making approach.

She is very straightforward and she hasn’t caused any troubles—she hasn’t broken any pain contracts. I have no reason to taper her off abruptly.^41^

And the best thing about it is that I involve the patient when I do this, because it’s not something secretive; it’s out in the open. So, I’ll say, “Okay, let’s pull this out, let’s print this [the pain contract] out.” And in fact I’ve given patients the copies of this.^50^

#### Subtheme 7.2: Long-Standing Patient–Provider Relationship and Trust Increased Willingness to Engage in Opioid Discussions (GRADE-CERQual Confidence Level: High)

Seven articles elucidated the impact of a long-standing patient–provider relationship on prescribing opioids among a sample of 195 providers (166 primary care, 4 nurse practitioners, 8 physician assistants, 8 internal medicine, 3 specialists, 2 osteopath, 2 pharmacists, 2 other).^34,39,40,42,44,50,52^ Having a long-standing relationship with a patient increased willingness to engage in discussions surrounding opioid prescribing. Providers noted that knowing the patient well, knowing their background, having a well-rounded level of trust, and having a strong relationship with their patient increased their comfort level when prescribing opioids:

“If you’re the family doctor or long-term psychiatrist or internist who’s known them [the patient] for 10 years and now they’ve developed a pain problem, you already have a bank of goodwill both ways.”^34^

### Theme 8: Interprofessional Support or Mentorship Improved Providers’ Willingness and Confidence to Engage in Patient Discussions Surrounding Opioid Prescribing Practices (GRADE-CERQual Confidence Level: Low)

Three articles that reported on 56 providers (46 primary care, 4 nurse practitioners, 2 physician assistants, 2 residents, 1 health psychologist, 1 other) explored the impact of mentorship on opioid prescribing.^48,53,59^ Primary care providers indicated that mentorship and support from specialists, experienced colleagues, or allied health professionals improved willingness and confidence to engage patients in discussions around prescribing opioids for CNCP. Advice on how to approach challenging patient cases, support from pharmacists on how to design opioid tapering plans, and validation from colleagues on the challenges of managing patients with CNCP were perceived as particularly beneficial.

[Group member] really walked me through with one patient in particular. … “Here is how I approach these patients: Tell me about your life; tell me about your day. Do you wake up in pain? Then what do you do?” And just those statements. … Here are the talking points.^53^

### Theme 9: Use of Prescription Drug Monitoring Programs for Risk Management Contributed to Providers’ Confidence in Opioid Prescribing and Discontinuation (GRADE-CERQual Confidence Level: Moderate)

Twelve studies that included 271 providers (192 primary care, 16 nurse practitioner, 10 physician assistant, 10 internal medicine, 9 specialists, 2 osteopath, 27 pharmacists, 2 other) reported on the impact of risk management tools on prescribing opioids.^33,35,36,39,40,47,49,50,54,55,58^ The availability of risk management tools for prescribing opioids, such as patient history, prescription drug monitoring programs (PDMPs), and urine drug testing, was perceived as beneficial and their implementation contributed to willingness to prescribe.

I had a patient who was getting oxycodone for six to eight months for a chronic pain issue … because we were concerned about his behavior, we did a urine tox which showed no oxycodone and, instead, showed morphine could’ve been heroin or anything he bought. So, we discontinued oxycodone.^33^

Providers also noted that risk management tools helped to better manage medication and prevent opioid abuse and diversion. For example, providers felt that treatment agreements set clear expectations about opioid use that could lead to meaningful discussions when risk was present:

“Okay, it’s clear to us that you’re not following through with the guidelines of the contract. And if that’s the case, then … I don’t feel comfortable prescribing for you anymore because you’re using in a way that’s unsafe.”^58^

## Mixed

### Theme 10: External Policies and Tools Increased Providers Confidence in Decisions to Prescribe or Discontinue Opioids. Meanwhile, External Pressures Decreased Willingness to Prescribe

#### Subtheme 10.1: Providers Adopted a Defensive Stance When Prescribing Opioids (GRADE-CERQual Confidence Level: Moderate)

Nine articles that included on 172 providers (101 primary care, 16 nurse practitioners, 9 physician assistants, 10 residents, 18 internal medicine, 12 specialist, 6 other) reported that it is advantageous for a provider to adopt a “defensive stance” (i.e., a position of distrust) when entering a patient–provider relationship that centers around prescribing opioids.^33,34,40,41,43,44,46,47,52^ This defensive stance likely arises from vigilance toward the identification of “red flags” for the prescription of opioids, such as risk of addiction:

“You become on guard right away. Whether rightfully or wrongfully, maybe to some extent, rightfully. But you right away think, okay, it’s not just like my average, easy visit. This is going to be something that I’ve got to be more challenging and more aware.”^52^

Few providers^47^ believe that it is their responsibility to be truly trusting of patient reports of pain. Rather, the majority of providers report skepticism or suspicion of patient motives, resulting in the adoption of a position of mistrust and a hesitancy to prescribe opioids.

#### Subtheme 10.2: Concern over Regulatory Scrutiny Decreased Willingness to Prescribe Opioids (GRADE-CERQual Confidence Level: Moderate)

Five articles reported on the impact that concern over regulatory scrutiny had on prescribing opioids among a sample of 70 providers (48 primary care, 16 nurse practitioners, 2 internal medicine, 3 specialists, 1 other).^34,43,45,50,51^ Concern over regulatory scrutiny or being held liable for a patient’s overdose or death led to a reduced willingness of general practitioners to prescribe opioids for CNCP.

If you have someone on a narcotic long term, it gets people’s attention even if it is justified, so I feel like I am under the microscope when I prescribe those drugs.^43^

There’s always a lurking fear … certainly someone could overdose on what I prescribed and then their family member could try to press charges.^45^

Some providers have witnessed colleagues lose their jobs over opioid prescribing and felt the need to protect their professional licensure, which led to a lower likelihood of prescribing opioids:

“… And doctors have lost their jobs, on my ward, over that issue.”^34^

#### Subtheme 10.3: Institutional Policies Reduced Subjectivity of Prescribing Opioids and Were Perceived as Beneficial (GRADE-CERQual Confidence Level: Moderate)

Four articles^43,47,58,59^ reported on the benefit of having institutional policies for prescribing opioids for CNCP among a sample of 56 providers (36 general practitioners, 17 nurse practitioners, 3 physician assistants).^33,37,48,49^ The presence of institutional policies for prescribing opioids or an institutional opioid committee was viewed as a beneficial framework that reduced subjectivity in prescribing, allowed for assertive communication with clients, and reduced the likelihood of patients becoming agitated with the provider when changes were made to opioid prescriptions.

“It doesn’t have to do with me, this is the policy of the clinic,” which, for those of us like myself who find it a little hard to say no, gives me a little stronger backing when I feel like I do need to cut someone off [discontinue opioids]. I don’t have to own that decision alone. …^47^

### Theme 11: Some Providers Struggled to Enact Guidelines, Especially among Complex Patients. Contrarily, Some Providers Felt the Guidelines Were Helpful for Making Decisions and Setting Boundaries (GRADE-CERQual Confidence Level: Moderate)

Five articles highlighted differing views on guidelines for prescribing opioids for CNCP among a sample of 74 providers (63 primary care, 4 nurse practitioners, 7 specialists). Providers expressed that practice guidelines for prescribing opioids for CNCP were diffuse, required interpretation, and were difficult to enact with complex patients.^37,50,52,55,58^ Most providers felt that guidelines were not straightforward and did not pertain to complex patients. Others felt that the guidelines had a great volume of information but were difficult to interpret, resulting in difficulty enacting recommendations within their clinic:

“I felt like I was somewhat flying by the seat of my pants with prescribing opioids. I was not generous by any means. I tried to be very careful. I tried to keep it limited. I still had people that were taking them and diverting, and didn’t feel like I had any guidelines to go by.”^50^

Some providers expressed gratitude for the guidelines, indicating that they enabled helpful decision-making processes and helped set effective boundaries. As described by Chang et al.^37^:

“Opioid prescribing guidelines enable helpful decision making processes when assessing risk, initiating opioids, or managing opioids. Guidelines help set effective boundaries (e.g., safe maximum dose, decision to taper or discontinue).”

## Interpretation

### Uncertainty about the Subjective Experience of Pain and Privileging Functional Outcomes

Pain is a complex and subjective experience that poses several measurement challenges. Currently, there exists no valid and reliable method of objectively quantifying the experience of pain. Uncertainty about the veracity of patient self-reports of pain was associated with diagnostic ambiguity and a reduced willingness to prescribe opioids. Until more objective physiologic/neurologic measurement techniques are perfected, clinicians who study pain will continue to rely on the careful use of established self-report measures of pain and its impacts. Privileging functional outcomes and active engagement in chronic pain self-management appeared to offset this ambiguity.

### Concerns over Patient and Community Safety and Regulatory Scrutiny

Providers reported concerns over patient adverse effects, physical tolerance, and addiction. Moreover, experiencing or witnessing negative patient-related salient events (e.g., patients who have overdosed) and provider-related salient events (e.g., threat to provider) contributed to hesitancy surrounding prescribing opioids to manage pain. Salient negative events can result in cognitive biases that impact the delivery of medical care,^61,62^ such as availability^63^ and representativeness biases.^64^ Cognitive behavioral techniques could be beneficial for highlighting cognitive biases. For example, cognitive restructuring could be used to acknowledge distorted thoughts and promote reasoned practice.^65^ Incorporating cognitive bias awareness into the curriculum at medical centers has yielded promising results, demonstrating that residents were able to recognize biases and create strategies to avoid biased reasoning.^66–68^ Given associations between chronic opioid use and an increased risk for opioid use disorder, overdose, and death,^69–72^ it is difficult to interpret whether concerns over adverse effects represent an accurate appreciation for potential opioid-related harms or relative risk aversion.

Providers reported that concern over drug diversion was a barrier to prescribing opioids for CNCP. Population-based estimates indicate that the prevalence of diversion within the community is 5%,^73^ with rates of diversion increasing as opioid prescribing rates increase.^74^ Reasons for opioid diversion vary, ranging from unawareness of the consequences of opioid diversion,^75^ to poor education about proper storage and disposal of opioids,^76^ to aberrant medication behaviors or misuse.^77^ Several strategies have been recommended to reduce drug diversion and identify those at risk of diverting, including (1) education, (2) urine screening, (3) patient contracts, (4) low dose initiation, (5) gradual dose titration, (6) use of PDMPs, and (7) completing multidimensional assessments with patients before prescribing opioids.^78–80^ It is unclear whether these interventions have a direct impact on reducing opioid diversion.^78^

Moderate confidence can be placed in the observation that concern over regulatory scrutiny was a barrier to prescribing opioids for CNCP. Though regulatory scrutiny and sanction can be effective methods for managing opioid prescribing behavior, regulatory policies vary by province, region, territory, state, and country and can result in barriers to prescribing,^81^ as well as unintended consequences, such as patients not receiving necessary prescriptions and seeking illicit opioids.^82^

### Long-Standing Relationships with Patients, Trust, and the Working Alliance

Moderate confidence can be placed in the observations that the patient–provider working alliance was a robust facilitator of opioid prescribing and appeared to center around trust and safety. Providers reported that patients’ withholding or minimizing information (e.g., history of substance use) reduced willingness to prescribe,^34,36,40^ whereas open communication and a trusting relationship facilitated opioid prescribing.^39,41^ Long-standing patient–provider relationships and shared decision making can reduce the impact of this complexity. Research suggests that physicians demonstrating compassion, having a strong working alliance, and portraying a patient-centered approach that involves patients in treatment decision making improve provider clinical decision making, positive communication, patient health, and treatment adherence.^83,84^ Punwasi et al. also observed that long-standing patient–provider relationships increase general practitioners’ confidence in prescription decisions.^85^ The adoption of such an empathic therapeutic approach may be particularly pertinent when prescribing opioids given that providers found conversations surrounding opioids to be difficult and potentially detrimental to the working alliance.

### Education, Time Constrains, and Availability of Nonopioid Alternatives

Moderate confidence can be placed in the observation that primary care providers are reluctant to prescribe opioids for CNCP due to insufficient formal education on opioid prescribing. Canadian medical undergraduate education programs offered an average of 16 h on pain management in 2007,^86^ and estimates suggest that only 20% of physicians in the United States received training about recognizing drug diversion or identifying signs of substance use disorders while in medical school.^87^ Health care providers also reported a lack of confidence in their ability to prescribe opioids to manage CNCP, specifically when no medical explanation for pain is present^35,51^ and in the presence of comorbidities (e.g., substance use disorder or mental illness).^33,48^ Education and training in chronic pain are necessary to enhance provider knowledge, confidence, and self-efficacy surrounding chronic pain management, opioid prescribing, and identifying drug abuse and addiction among people with chronic pain.^88,89^

Moderate confidence can be placed in the observation that a lack of readily accessible nonopioid alternatives (e.g., referral pathways, community resources) was a facilitator to prescribing opioids. A similar theme was extracted from a previous synthesis of qualitative literature on prescribing opioids for chronic pain.^85^ Insufficient nonopioid alternative treatments can result in protracted wait times, which is problematic given that wait times for chronic pain care in excess of 6 months have been deemed medically unacceptable.^90^ As evidence for this, more than 50% of patients referred to chronic pain clinics in Canada are not admitted within the recommended wait time, with many waiting years for admittance, a circumstance that has not changed over the last 10 years.^91^ Canadian guidelines for opioid therapy in CNCP highly recommend maximizing the use of nonopioid therapies.^12^ Yet, access to such therapies is difficult due to long wait list times, few multidisciplinary support programs available,^91^ a lack of digital platforms for chronic pain self-management,^92^ and lack of access to publicly funded allied health professionals (e.g., physiotherapy, psychology).^93^

Moderate confidence can be placed in the observation that brief appointment durations are a barrier to prescribing opioids for pain management. The management of chronic diseases often requires more time than physicians have available, which is a significant obstacle to the delivery of quality care.^94^ This is interesting given that a recent cross-sectional study of physician behavior in primary care reported that opioid prescriptions increased by 33% as the workday progressed and by 17% when appointments ran behind schedule.^95^ It would appear that brief appointments in combination with a perceived lack of availability to discuss nonopioid therapies influence clinical decision making when prescribing opioids.

### Relationship between the Current Synthesis and the COVID-19 Pandemic

It is important to note that we synthesized evidence published before the global COVID-19 pandemic. The COVID-19 pandemic had a clear impact on pain management globally, including prescribing opioids for CNCP. The COVID-19 pandemic introduced additional considerations for prescribers when weighing the potential risks and benefits associated with prescribing opioids (e.g., opioids can increase the risk of respiratory depression, suppress the immune system, or mask fever and myalgias that may be related to COVID-19).^96,97^ Further, providers were forced to transition to telemedicine, which imposed additional challenges to assessment and maintaining strong therapeutic relationships.^96,97^ Unfortunately, there is insufficient evidence to appreciate the full impact of the COVID-19 pandemic on the prescription of opioids for CNCP or whether the impact will be transient or long-lasting. We contend that a strong appreciation of the barriers and facilitators for prescribing opioids for CNCP before the COVID-19 pandemic will aid in our understanding of how prescribing practices have evolved during and after the pandemic.

## Strengths of This Review and Reflexivity

This review was undertaken by a multidisciplinary team with diverse viewpoints and experiences in chronic pain management, including two practicing clinical psychologists with expertise in chronic pain management, one practicing anesthesiologist with expertise in chronic pain management, and two trainees (one experimental and one clinical) from the discipline of psychology. Members of our team engage in regular clinical practice and have clinical experience working with people who live with pain and use opioids with benefits and opioid-related harms. Members of our team are involved in federal mentorship programs for chronic pain, substance use, and addictions and co-developed the safe prescribing course for opioids. As such, we likely tended to identify and code potentially modifiable barriers and facilitators that coincide with evidence and theory on the adoption of recommendations made by clinical practice guidelines. It is also important to note that we coded barriers and facilitators based on the likelihood of decreasing or increasing the frequency of an event, respectively. There was no value judgment placed on the appropriateness of such deviations to clinical practice. This is one of the first reviews to qualitatively identify health care providers’ perceived barriers and facilitators when prescribing opioids for patients with CNCP. This synthesis will serve as a starting point when understanding potential changes in barriers and facilitators of opioid prescribing that may have occurred since the COVID-19 pandemic.

## Limitations of This Review

First, we focused on evidence from North America. The results of this synthesis may not extend to other geographic regions given the sociopolitical nature of the opioid crisis. Second, studies that focused on opioid use disorder and the prescription of opioid agonist and antagonist treatments were excluded. Thus, results are not generalizable to the prescription of opioid agonist treatments. Third, this review focused on prescribing opioids rather than the alignment of prescribing with practice guidelines due to a lack of available literature. As such, some facilitators may represent barriers to appropriate prescribing. For example, a lack of opioid alternatives facilitates opioid prescribing yet can be a barrier to optimizing nonpharmacological care prior to prescribing opioids for CNCP.

## Conclusion

Providers have an ethical responsibility to work with patients to manage CNCP, and such management may include chronic opioid therapy. The current synthesis identified barriers to prescribing opioids, including providers’ uncertainty regarding the subjectivity of pain and efficacy of opioids, concern for patient and community safety, past negative experiences, and fear of harming the therapeutic alliance. Facilitators to prescribing opioids included prioritizing improvement in patient function, a strong therapeutic alliance, and interprofessional mentorship and support. Our findings also revealed complex processes that could act as barriers or facilitators to prescribing opioids for CNCP, such as external policies, opioid prescribing guidelines, and organizational constraints. Suggestions for improving judicious opioid prescribing include continuing education, mentoring or support from experts, and ensuring that regulators apply scrutiny consistent with best evidence.

## References

[1] Volkow ND, McLellan AT. Opioid abuse in chronic pain–misconceptions and mitigation strategies. N Engl J Med. 2016;374(13):1253–28. doi:10.1056/NEJMra1507771. 27028915

[2] Breivik H, Collett B, Ventafridda V, Cohen R, Gallacher D. Survey of chronic pain in Europe: prevalence, impact on daily life, and treatment. Eur J Pain. 2006;10(4):287–333. doi:10.1016/j.ejpain.2005.06.009. 16095934

[3] Nahin RL. Estimates of pain prevalence and severity in adults: United States, 2012. J Pain. 2015;16(8):769–80. doi:10.1016/j.jpain.2015.05.002. 26028573 PMC4562413

[4] Rosenblum A, Marsch LA, Joseph H, Portenoy RK. Opioids and the treatment of chronic pain: controversies, current status, and future directions. Exp Clin Psychopharmacol. 2008;16(5):405–16. doi:10.1037/a0013628. 18837637 PMC2711509

[5] Kolodny A, Courtwright DT, Hwang CS, Kreiner P, Eadie JL, Clark TW, Alexander GC. The prescription opioid and heroin crisis: a public health approach to an epidemic of addiction. Annu Rev Public Health. 2015;36:559–74. doi:10.1146/annurev-publhealth-031914-122957. 25581144

[6] Centers for Disease Control and Prevention. Vital signs: overdoses of prescription opioid pain relievers—United States, 1999–2008. MMWR Morb Mortal Wkly Rep. 2011;60(43):1487. 22048730

[7] Drug Abuse Warning Network. Substance abuse treatment admissions by primary substance of abuse, according to sex, age group, race, and ethnicity: the DAWN report. 2013;800–729 https://www.samhsa.gov/data/sites/default/files/DAWN2k11ED/DAWN2k11ED/DAWN2k11ED.htm.

[8] Substance Abuse and Mental Health Services Administration, Drug Abuse Warning Network, 2011: National Estimates of Drug-Related Emergency Department Visits. HHS Publication No. (SMA) 13-4760, DAWN Series D-39. Rockville, MD: Substance Abuse and Mental Health Services Administration; 2013.

[9] Chen LH, Hedegaard H, Warner M. Drug-poisoning deaths involving opioid analgesics: United States, 1999-2011. NCHS data brief 2014. 2014;(166):1–8. 25228059

[10] Busse JW, Wang L, Kamaleldin M, Craigie S, Riva JJ, Montoya L, Mulla SM, Lopes LC, Vogel N, Chen E, et al. Opioids for chronic noncancer pain: a systematic review and meta-analysis. JAMA. 2018;320(23):2448–60. doi:10.1001/jama.2018.18472. 30561481 PMC6583638

[11] Dowell D, Haegerich TM, Chou R. CDC guideline for prescribing opioids for chronic pain—United States, 2016. JAMA. 2016;315(15):1624–45. doi:10.1001/jama.2016.1464. 26977696 PMC6390846

[12] Busse JW, Craigie S, Juurlink DN, Buckley DN, Wang L, Couban RJ, Agoritsas T, Akl EA, Carrasco-Labra A, Cooper L, et al. Guideline for opioid therapy and chronic noncancer pain. CMAJ. 2017;189(18):E659–66. doi:10.1503/cmaj.170363. 28483845 PMC5422149

[13] Clarke H, Bao J, Weinrib A, Dubin RE, Kahan M. Canada’s hidden opioid crisis: the health care system’s inability to manage high-dose opioid patients: fallout from the 2017 Canadian opioid guidelines. Can Fam Physician. 2019;65:612–14. 31515308 PMC6741787

[14] Furlan AD, Williamson OD. New Canadian guidance on opioid use for chronic pain: necessary but not sufficient. CMAJ. 2017;189(18):E650–E1. doi:10.1503/cmaj.170431. 28483843 PMC5422147

[15] Lynch ME, Katz J. “One size fits all” doesn’t fit when it comes to long-term opioid use for people with chronic pain. Can J Pain. 2017;1(1):2–7. doi:10.1080/24740527.2017.1319733. 35005336 PMC8730555

[16] Gallagher R, Hatcher L. Will the new opioid guidelines harm more people than they help? Yes. Can Fam Physician. 2018;64:101–02. 29449237 PMC5964381

[17] Cicero TJ, Ellis MS, Surratt HL, Kurtz SP. The changing face of heroin use in the United States: a retrospective analysis of the past 50 years. JAMA psychiatry. 2014;71(7):821–26. doi:10.1001/jamapsychiatry.2014.366. 24871348

[18] Belzak L, Halverson J. The opioid crisis in Canada: a national perspective. Health Promot Chronic Dis Prev Can. 2018;38(6):224–33. doi:10.24095/hpcdp.38.6.02. 29911818 PMC6034966

[19] Federal, provincial, and territorial Special Advisory Committee on the Epidemic of Opioid Overdoses. Opioid- and stimulant-related harms in Canada. Ottawa (Ontario, Canada): Public Health Agency of Canada; 2022 Mar.

[20] Toye F, Seers K, Tierney S, Barker KL. A qualitative evidence synthesis to explore healthcare professionals’ experience of prescribing opioids to adults with chronic non-malignant pain. BMC Fam Pract. 2017;18(1):94. doi:10.1186/s12875-017-0663-8. 29178843 PMC5702226

[21] Moher D, Liberati A, Tetzlaff J, Altman DG. Preferred reporting items for systematic reviews and meta-analyses: the PRISMA statement. Ann Intern Med. 2009;151(4):264–69. doi:10.7326/0003-4819-151-4-200908180-00135. 19622511

[22] Tong A, Flemming K, McInnes E, Oliver S, Craig J. Enhancing transparency in reporting the synthesis of qualitative research: ENTREQ. BMC Med Res Methodol. 2012;12:181. doi:10.1186/1471-2288-12-181. 23185978 PMC3552766

[23] Rash JA, Buckley N, Busse JW, Campbell TS, Corace K, Cooper L, Flusk D, Iorio A, Lavoie KL, Poulin PA, et al. Healthcare provider knowledge, attitudes, beliefs, and practices surrounding the prescription of opioids for chronic non-cancer pain in North America: protocol for a mixed-method systematic review. Syst Rev. 2018;7(1):Article number 189. doi:10.1186/s13643-018-0858-7. PMC6234680 30424800

[24] Fischer B, Keates A, Buhringer G, Reimer J, Rehm J. Non-medical use of prescription opioids and prescription opioid-related harms: why so markedly higher in North America compared to the rest of the world? Addiction. 2014;109(2):177–81. doi:10.1111/add.12224. 23692335

[25] Ouzzani M, Hammady H, Fedorowicz Z, Elmagarmid A. Rayyan — a web and mobile app for systematic reviews. Systematic Reviews. 2016;5:210. doi:10.1186/s13643-016-0384-4. PMC5139140 27919275

[26] Malterud K. Qualitative metasynthesis: a research method for medicine and health sciences. New York (NY): Routledge; 2019.

[27] Thomas J, Harden A. Methods for the thematic synthesis of qualitative research in systematic reviews. BMC Med Res Methodol. 2008;8:45. doi:10.1186/1471-2288-8-45. 18616818 PMC2478656

[28] Atkins L, Francis J, Islam R, O’Connor D, Patey A, Ivers N, Foy R, Duncan EM, Colquhoun H, Grimshaw JM, et al. A guide to using the theoretical domains framework of behaviour change to investigate implementation problems. Implement Sci. 2017;12(1):77. doi:10.1186/s13012-017-0605-9. 28637486 PMC5480145

[29] Cane J, O’Connor D, Michie S. Validation of the theoretical domains framework for use in behaviour change and implementation research. Implement Sci. 2012;7:37. doi:10.1186/1748-5908-7-37. 22530986 PMC3483008

[30] Critical Appraisal Skills Programme. CASP qualitative checklist; 2018 [accessed 2018 Mar 25]. https://casp-uk.net/wp-content/uploads/2018/01/CASP-Qualitative-Checklist-2018.pdf.

[31] Noyes J, Booth A, Flemming K, Garside R, Harden A, Lewin S, Pantoja T, Hannes K, Cargo M, Thomas J. Cochrane qualitative and implementation methods group guidance paper 3: methods for assessing methodological limitations, data extraction and synthesis, and confidence in synthesized qualitative findings. J Clin Epidemiol. 2018;97:49–58. doi:10.1016/j.jclinepi.2017.06.020. 29247700

[32] Thomas J, Harden A, Oakley A, Oliver S, Sutcliffe K, Rees R, Brunton G, Kavanagh J. Integrating qualitative research with trials in systematic reviews. BMJ. 2004;328(7446):1010–12. doi:10.1136/bmj.328.7446.1010. 15105329 PMC404509

[33] Barry DT, Irwin KS, Jones ES, Becker WC, Tetrault JM, Sullivan LE, Hansen H, O’Connor PG, Schottenfeld RS, Fiellin DA. Opioids, chronic pain, and addiction in primary care. J Pain. 2010;11(12):1442–50. doi:10.1016/j.jpain.2010.04.002. 20627817 PMC2955997

[34] Buchman DZ, Ho A, Illes J. You present like a drug addict: patient and clinician perspectives on trust and trustworthiness in chronic pain management. Pain Med. 2016;17(8):1394–406. doi:10.1093/pm/pnv083. 26759389 PMC4975016

[35] Calcaterra SL, Drabkin AD, Leslie SE, Doyle R, Koester S, Frank JW, Reich JA, Binswanger IA. The hospitalist perspective on opioid prescribing: a qualitative analysis. J Hosp Med. 2016;11(8):536–42. doi:10.1002/jhm.2602. 27157317 PMC4970927

[36] Chang JS, Kushel M, Miaskowski C, Ceasar R, Zamora K, Hurstak E, Knight KR. Provider experiences with the identification, management, and treatment of co-occurring chronic noncancer pain and substance use in the safety net. Subst Use Misuse. 2017;52(2):251–55. doi:10.1080/10826084.2016.1223138. 27754719 PMC5345572

[37] Chang Y, Zhu KL, Florez ID, Cho SM, Zamir N, Toma A, Mirza RD, Guyatt GH, Buckley N, Busse JW. Attitudes toward the Canadian guideline for safe and effective use of opioids for chronic non-cancer pain: a qualitative study. J Opioid Manag. 2016;12(6):377–87. doi:10.5055/jom.2016.0357. 28059430

[38] Clark LG, Upshur CC. Family medicine physicians’ views of how to improve chronic pain management. J Am Board Fam Med. 2007;20(5):479–82. doi:10.3122/jabfm.2007.05.070029. 17823465

[39] Click IA, Basden JA, Bohannon JM, Anderson H, Tudiver F. Opioid prescribing in rural family practices: a qualitative study. Subst Use Misuse. 2018;53(4):533–40. doi:10.1080/10826084.2017.1342659. 28857643

[40] Elder NC, Simmons T, Regan S, Gerrety E. Care for patients with chronic nonmalignant pain with and without chronic opioid prescriptions: a report from the Cincinnati area research group (CARinG) network. J Am Board Fam Med. 2012;25(5):652–60. doi:10.3122/jabfm.2012.05.120032. 22956700

[41] Esquibel AY, Borkan J. Doctors and patients in pain: conflict and collaboration in opioid prescription in primary care. Pain. 2014;155(12):2575–82. doi:10.1016/j.pain.2014.09.018. 25261714

[42] Fischer MA, McKinlay JB, Katz JN, Gerstenberger E, Trachtenberg F, Marceau LD, Welch LC. Physician assessments of drug seeking behavior: a mixed methods study. PLoS One. 2017;12(6):e0178690. doi:10.1371/journal.pone.0178690. 28644835 PMC5482434

[43] Fontana JS. The social and political forces affecting prescribing practices for chronic pain. J Prof Nurs. 2008;24(1):30–35. doi:10.1016/j.profnurs.2007.06.002. 18206840

[44] Harle CA, Bauer SE, Hoang HQ, Cook RL, Hurley RW, Fillingim RB. Decision support for chronic pain care: how do primary care physicians decide when to prescribe opioids? A qualitative study. BMC Family Practice. 2015;16:48. doi:10.1186/s12875-015-0264-3. 25884340 PMC4399157

[45] Hurstak EE, Kushel M, Chang J, Ceasar R, Zamora K, Miaskowski C, Knight K. The risks of opioid treatment: perspectives of primary care practitioners and patients from safety-net clinics. Subst Abus. 2017;38(2):213–21. doi:10.1080/08897077.2017.1296524. 28394752 PMC5568522

[46] Kennedy LC, Binswanger IA, Mueller SR, Levy C, Matlock DD, Calcaterra SL, Koester S, Frank JW. “Those conversations in my experience don’t go well”: a qualitative study of primary care provider experiences tapering long-term opioid medications. Pain Med. 2017. 10.1093/pm/pnx276 PMC6454789 29126138

[47] Knight KR, Kushel M, Chang JS, Zamora K, Ceasar R, Hurstak E, Miaskowski C. Opioid pharmacovigilance: a clinical-social history of the changes in opioid prescribing for patients with co-occurring chronic non-cancer pain and substance use. Soc Sci Med. 2017;186:87–95. doi:10.1016/j.socscimed.2017.05.043. 28599142 PMC5551446

[48] Liddy C, Smyth C, Poulin PA, Joschko J, Sheppard M, Keely E. Supporting better access to chronic pain specialists: the Champlain BASE TM eConsult service. J Am Board Fam Med. 2017;30(6):766–74. doi:10.3122/jabfm.2017.06.170170. 29180551

[49] Penney LS, Ritenbaugh C, DeBar LL, Elder C, Deyo RA. Provider and patient perspectives on opioids and alternative treatments for managing chronic pain: a qualitative study. BMC Fam Pract. 2017;17(1):Article 164. doi:10.1186/s12875-016-0566-0. PMC5390355 28403822

[50] Robertson A, Hitzig SL, Furlan AD. An evaluation of the performance of the opioid manager clinical tool in primary care: a qualitative study. J Opioid Manag. 2014;10(3):187–99. doi:10.5055/jom.2014.0207. 24944069

[51] Spitz A, Moore AA, Papaleontiou M, Granieri E, Turner BJ, Reid MC. Primary care providers’ perspective on prescribing opioids to older adults with chronic non-cancer pain: a qualitative study. BMC Geriatrics. 2011;11:35. doi:10.1186/1471-2318-11-35. 21752299 PMC3212901

[52] Desveaux L, Saragosa M, Kithulegoda N, Ivers NM. Understanding the behavioural determinants of opioid prescribing among family physicians: a qualitative study. BMC Fam Pract. 2019;20(1):59. doi:10.1186/s12875-019-0947-2. 31077137 PMC6511163

[53] Hulen E, Saha S, Morasco BJ, Zeigler C, Mackey K, Edwards ST. Sources of distress in primary care opioid management and the role of a controlled substance review group: a qualitative study. Pain Med. 2018;19(8):1570–77. doi:10.1093/pm/pnx259. 29099982

[54] Kang I, Urick B, Vohra R, Ives TJ. Physician-pharmacist collaboration on chronic non-cancer pain management during the opioid crisis: a qualitative interview study. Res Social Adm Pharm. 2019;15(8):1027–31. doi:10.1016/j.sapharm.2019.04.052. 31053466

[55] Militello LG, Anders S, Downs SM, Diiulio J, Danielson EC, Hurley RW, Harle CA. Understanding how primary care clinicians make sense of chronic pain. Cogn Technol Work. 2018;20(4):575–84. doi:10.1007/s10111-018-0491-1. 30842708 PMC6398613

[56] Satterwhite S, Knight KR, Miaskowski C, Chang JS, Ceasar R, Zamora K, Kushel M. Sources and impact of time pressure on opioid management in the safety-net. J Am Board Fam Med. 2019;32(3):375–82. doi:10.3122/jabfm.2019.03.180306. 31068401 PMC6988512

[57] Tong ST, Hochheimer CJ, Brooks EM, Sabo RT, Jiang V, Day T, Rozman JS, Kashiri PL, Krist AH. Chronic opioid prescribing in primary care: factors and perspectives. Ann Fam Med. 2019;17(3):200–06. doi:10.1370/afm.2357. 31085523 PMC6827634

[58] Wyse JJ, Ganzini L, Dobscha SK, Krebs EE, Morasco BJ. Setting expectations, following orders, safety, and standardization: clinicians’ strategies to guide difficult conversations about opioid prescribing. J Gen Intern Med. 2019;34(7):1200–06. doi:10.1007/s11606-019-04983-y. 31011964 PMC6614300

[59] Wyse JJ, Ganzini L, Dobscha SK, Krebs EE, Zamudio J, Morasco BJ. Clinical strategies for the treatment and management of patients prescribed long-term opioid therapy. Pain Medicine. 2018;20(9):1737–1744. doi:10.1093/pm/pny211. 30388259

[60] Berger R. Now I see it, now I don’t: researcher’s position and reflexivity in qualitative research. Qual Res. 2015;15(2):219–34. doi:10.1177/1468794112468475.

[61] Blumenthal-Barby JS, Krieger H. Cognitive biases and heuristics in medical decision making: a critical review using a systematic search strategy. Med decis making. 2015;35(4):539–57. doi:10.1177/0272989X14547740. 25145577

[62] Saini V, Garcia-Armesto S, Klemperer D, Paris V, Elshaug AG, Brownlee S, Ioannidis JPA, Fisher ES. Drivers of poor medical care. Lancet. 2017;390(10090):178–90. doi:10.1016/S0140-6736(16)30947-3. 28077235

[63] Cioffi J. A study of the use of past experiences in clinical decision making in emergency situations. Int J Nurs Stud. 2001;38(5):591–99. doi:10.1016/S0020-7489(00)00096-1. 11524105

[64] Hall KH. Reviewing intuitive decision-making and uncertainty: the implications for medical education. Med Edu. 2002;36(3):216–24. doi:10.1046/j.1365-2923.2002.01140.x. 11879511

[65] McCaffrey R, Zerwekh J, Keller K. Pain management: cognitive restructuring as a model for teaching nursing students. Nurse Educ. 2005;30(5):226–30. doi:10.1097/00006223-200509000-00012. 16170266

[66] Graber ML, Trowbridge R, Myers JS, Umscheid CA, Strull W, Kanter MH. The next organizational challenge: finding and addressing diagnostic error. Jt Comm J Qual Patient Saf. 2014;40(3):102–10. doi:10.1016/s1553-7250(14)40013-8. 24730205

[67] Ogdie AR, Reilly JB, Pang WG, Keddem S, Barg FK, Von Feldt JM, Myers JS. Seen through their eyes: residents’ reflections on the cognitive and contextual components of diagnostic errors in medicine. Acad med. 2012;87(10):1361–67. doi:10.1097/ACM.0b013e31826742c9. 22914511 PMC3703642

[68] Royce CS, Hayes MM, Schwartzstein RM. Teaching critical thinking: a case for instruction in cognitive biases to reduce diagnostic errors and improve patient safety. Acad med. 2019;94(2):187–94. doi:10.1097/ACM.0000000000002518. 30398993

[69] Chou R, Turner JA, Devine EB, Hansen RN, Sullivan SD, Blazina I, Dana T, Bougatsos C, Deyo RA. The effectiveness and risks of long-term opioid therapy for chronic pain: a systematic review for a national institutes of health pathways to prevention workshop. Ann Intern Med. 2015;162(4):276–86. doi:10.7326/M14-2559. 25581257

[70] Gomes T, Juurlink DN, Dhalla IA, Mailis-Gagnon A, Paterson JM, Mamdani MM. Trends in opioid use and dosing among socio-economically disadvantaged patients. Open Med. 2011;5(1):e13–22. doi:10.1016/j.jpain.2008.10.008. 22046214 PMC3205807

[71] Gomes T, Tadrous M, Mamdani MM, Paterson JM, Juurlink DN. The burden of opioid-related mortality in the United States. JAMA netw open. 2018;1(2):e180217. doi:10.1001/jamanetworkopen.2018.0217. 30646062 PMC6324425

[72] Juurlink DN, Dhalla IA. Dependence and addiction during chronic opioid therapy. J Med Toxicol. 2012;8(4):393–99. doi:10.1007/s13181-012-0269-4. 23073725 PMC3550262

[73] Han B, Compton WM, Jones CM, Cai R. Nonmedical prescription opioid use and use disorders among adults aged 18 through 64 years in the United States, 2003-2013. JAMA. 2015;314(14):1468–78. doi:10.1001/jama.2015.11859. 26461997

[74] Volkow ND, Jones EB, Einstein EB, Wargo EM. Prevention and treatment of opioid misuse and addiction: a review. JAMA psychiatry. 2019;76(2):208–16. doi:10.1001/jamapsychiatry.2018.3126. 30516809

[75] Johnston LD, O’Malley PM, Miech RA, Bachman JG, Schulenberg JE. Monitoring the future national survey results on drug use, 1975-2015: overview, key findings on adolescent drug use. Ann Arbor (MI, USA): Institute for Social Research, The University of Michigan; 2016.

[76] Kennedy-Hendricks A, Gielen A, McDonald E, McGinty EE, Shields W, Barry CL. Medication sharing, storage, and disposal practices for opioid medications among US adults. JAMA Intern Med. 2016;176(7):1027–29. doi:10.1001/jamainternmed.2016.2543. 27295629

[77] Hulme S, Bright D, Nielsen S. The source and diversion of pharmaceutical drugs for non-medical use: a systematic review and meta-analysis. Drug Alcohol Depend. 2018;186:242–56. doi:10.1016/j.drugalcdep.2018.02.010. 29626777

[78] Canadian Agency for Drugs and Technologies in Health (CADTH). Opioid management practices for the prevention of drug diversion and misuse: a review of the clinical evidence and guidelines. Ottawa (Canada): CADTH. Rapid response review; 2012.

[79] Fischer B, Argento E. Prescription opioid related misuse, harms, diversion and interventions in Canada: a review. Pain Physician. 2012;15(3 Suppl):ES191–203. doi:10.36076/ppj.2012/15/ES191. 22786457

[80] Volkow ND, McLellan TA. Curtailing diversion and abuse of opioid analgesics without jeopardizing pain treatment. JAMA. 2011;305(13):1346–47. doi:10.1001/jama.2011.369. 21467287

[81] Vranken MJ, Lisman JA, Mantel-Teeuwisse AK, Jünger S, Scholten W, Radbruch L, Payne S, Schutjens MH. Barriers to access to opioid medicines: a review of national legislation and regulations of 11 central and Eastern European countries. Lancet Oncol. 2016;17(1):e13–22. doi:10.1016/S1470-2045(15)00365-4. 26758755

[82] Furlan AD, Carnide N, Irvin E, Van Eerd D, Munhall C, Kim J, Li CMF, Hamad A, Mahood Q, MacDonald S. A systematic review of strategies to improve appropriate use of opioids and to reduce opioid use disorder and deaths from prescription opioids. Can J Pain. 2018;2(1):218–35. doi:10.1080/24740527.2018.1479842. 35005381 PMC8730669

[83] Joosten EA, DeFuentes-Merillas L, de Weert GH, Sensky T, van der Staak CP, de Jong CA. Systematic review of the effects of shared decision-making on patient satisfaction, treatment adherence and health status. Psychother Psychosom. 2008;77(4):219–26. doi:10.1159/000126073. 18418028

[84] Matthias MS. Opioid tapering and the patient-provider relationship. J Gen Intern Med. 2020;35(1):8–9. doi:10.1007/s11606-019-05337-4. 31705464 PMC6957641

[85] Punwasi R, de Kleijn L, Rijkels-Otters JBM, Veen M, Chiarotto A, Koes B. General practitioners’ attitudes towards opioids for non-cancer pain: a qualitative systematic review. BMJ open. 2022;12(2):e054945. doi:10.1136/bmjopen-2021-054945. PMC8808445 35105588

[86] Watt-Watson J, McGillion M, Hunter J, Choiniere M, Clark AJ, Dewar A, Johnston C, Lynch M, Morley-Forster P, Moulin D, et al. A survey of prelicensure pain curricula in health science faculties in Canadian universities. Pain Res Manag. 2009;14(6):439–44. doi:10.1155/2009/307932. 20011714 PMC2807771

[87] National Center on Addiction and Substance Abuse. Under the counter: the diversion and abuse of controlled prescription drugs in the US. New York (NY): Columbia University; 2005.

[88] Raheemullah A, Andruska N, Saeed M, Kumar P. Improving residency education on chronic pain and opioid use disorder: evaluation of CDC guideline-based education. Subst Use Misuse. 2020;55(4):684–90. doi:10.1080/10826084.2019.1691600. 31757179

[89] Vargovich AM, Schumann ME, Xiang J, Ginsberg AD, Palmer BA, Sperry JA. Difficult conversations: training medical students to assess, educate, and treat the patient with chronic pain. Acad Psychiatry. 2019;43(5):494–98. doi:10.1007/s40596-019-01072-4. 31168741

[90] Lynch ME, Campbell F, Clark AJ, Dunbar MJ, Goldstein D, Peng P, Stinson J, Tupper H. A systematic review of the effect of waiting for treatment for chronic pain. Pain. 2008;136(1–2):97–116. doi:10.1016/j.pain.2007.06.018. 17707589

[91] Choinière M, Peng P, Gilron I, Buckley N, Williamson O, Janelle-Montcalm A, Baerg K, Boulanger A, Di Renna T, Finley GA, et al. Accessing care in multidisciplinary pain treatment facilities continues to be a challenge in Canada. Reg Anesth Pain Med. 2020;45(12):943–48. doi:10.1136/rapm-2020-101935. 33024007

[92] Eccleston C, Blyth FM, Dear BF, Fisher EA, Keefe FJ, Lynch ME, Palermo TM, Reid MC, Williams ACC. Managing patients with chronic pain during the COVID-19 outbreak: considerations for the rapid introduction of remotely supported (eHealth) pain management services. Pain. 2020;161(5):889–93. doi:10.1097/j.pain.0000000000001885. 32251203 PMC7172975

[93] Lynch ME, Campbell FA, Clark AJ, Dunbar MJ, Goldstein D, Peng P, Stinson J, Tupper H. Waiting for treatment for chronic pain - a survey of existing benchmarks: toward establishing evidence-based benchmarks for medically acceptable waiting times. Pain Res Manag. 2007;12(4):245–48. doi:10.1155/2007/891951. 18080042 PMC2670734

[94] Ostbye T, Yarnall KS, Krause KM, Pollak KI, Gradison M, Michener JL. Is there time for management of patients with chronic diseases in primary care? Ann Fam Med. 2005;3(3):209–14. doi:10.1370/afm.310. 15928223 PMC1466884

[95] Neprash HT, Barnett ML. Association of primary care clinic appointment time with opioid prescribing. JAMA netw open. 2019;2(8):e1910373. doi:10.1001/jamanetworkopen.2019.10373. 31469396 PMC6724149

[96] Puntillo F, Giglio M, Brienza N, Viswanath O, Urits I, Kaye AD, Pergolizzi J, Paladini A, Varrassi G. Impact of COVID-19 pandemic on chronic pain management: looking for the best way to deliver care. Best Pract Res Clin Anaesthesiol. 2020;34(3):529–37. doi:10.1016/j.bpa.2020.07.001. 33004164 PMC7366114

[97] Shanthanna H, Strand NH, Provenzano DA, Lobo CA, Eldabe S, Bhatia A, Wegener J, Curtis K, Cohen SP, Narouze S. Caring for patients with pain during the COVID-19 pandemic: consensus recommendations from an international expert panel. Anaesthesia. 2020;75(7):935–44. doi:10.1111/anae.15076. 32259288 PMC7262200

